# Liver resection, radiofrequency ablation, and radiofrequency ablation combined with transcatheter arterial chemoembolization for very-early- and early-stage hepatocellular carcinoma: A systematic review and Bayesian network meta-analysis for comparison of efficacy

**DOI:** 10.3389/fonc.2022.991944

**Published:** 2022-10-28

**Authors:** Yunlong Zhang, Yunlong Qin, Peng Dong, Houfa Ning, Guangzhi Wang

**Affiliations:** ^1^ School of Medical Imaging, Weifang Medical University, Weifang, China; ^2^ Department of Medical Imaging Center, Affiliated Hospital of Weifang Medical University, Weifang Medical University, Weifang, China

**Keywords:** liver resection, radiofrequency ablation, transcatheter arterial chemoembolization, overall survival, recurrence-free survival, hepatocellular carcinoma

## Abstract

**Objective:**

To compare the efficacy of liver resection (LR), radiofrequency ablation (RFA), and radiofrequency ablation combined with transcatheter arterial chemoembolization (RFA+TACE) in the treatment of very-early- and early-stage hepatocellular carcinoma (HCC).

**Methods:**

We systemically searched the PubMed, Embase, and Cochrane Library databases. Randomized controlled trials (RCTs) and observational analyses with propensity score-matched cohort analyses (PSMs) comparing any two of the three treatments were included in this study. The primary result was overall survival (OS) and the secondary result was recurrence-free survival (RFS), which were analyzed by calculating the hazard ratio (HR) and 95% confidence intervals (CI).

**Results:**

A total of 25 studies (4249 patients), including 10 RCTs and 15 PSM observational studies, met the inclusion criteria. Although there was no significant difference between LR and RFA in terms of one-year OS, though LR showed superior performance for three- and five-year OS (at three years, HR: 0.74, 95% CI: 0.56-0.96; at five years, HR: 0.73, 95% CI: 0.55-0.94). In addition, significantly higher rates of RFS at one-, three- and five-year follow-up were found for LR than for RFA alone (at one year, HR: 0.68, 95% CI: 0.51-0.92; at three years, HR: 0.67, 95% CI: 0.55-0.81; at five years, HR: 0.61, 95% CI: 0.48-0.78). The combination of RFA+TACE was superior to RFA alone based on one-year RFS (HR: 0.57, 95% CI: 0.34-0.96), while there were no significant differences in OS at one, three, and five years, and in RFS at three and five years.

**Conclusions:**

For very-early- and early-stage HCC, this systematic review and network meta-analysis showed that the efficacy of LR is superior to that of RFA alone, regardless of whether the evaluation is based on either OS or RFS. The advantages of RFA+TACE compared to RFA alone are limited, and further studies are needed to determine whether combination therapy is necessary, i.e., results in significantly improved outcomes.

**Systematic Review Registration:**

The study was registered with http://www.crd.york.ac.uk/PROSPERO, identifier: CRD42022299269

## 1 Introduction

Hepatocellular carcinoma (HCC) is the main pathologic type of primary liver cancer. According to the data released by GLOBOCAN 2020, the number of new cases of primary liver cancer worldwide was as high as 906,000, ranking sixth of the most commonly diagnosed cancers, and the death toll was as high as 830,000, ranking third as a cause of cancer death (1). In general, tumor characteristics such as size, number, vascular invasion, liver function status, and patient functional status are major factors in clinical decision making (2). These factors also form the basis of the Barcelona Clinic Liver Cancer (BCLC) stages. In the 2022 update for the BCLC staging system, the very early stage (BCLC 0) is defined as a solitary HCC < 2cm, and the early stage (BCLC A) is defined as a solitary HCC irrespective of size or as a multifocal HCC with up to three nodules (none of them > 3 cm), without macrovascular invasion, extrahepatic spread, or cancer-related symptoms (PS-0). Liver resection (LR), ablation, and liver transplantation (LT) are recommended as radical treatments for very-early- and early-stage (BCLC 0/A) patients (3).

LR and LT represent the first treatment options in patients with early tumors from an intention-to-treat perspective (4). However, these options are limited due to potential liver insufficiency, portal hypertension and related diseases, and organ shortages. Therefore, it is essential to seek an effective alternative therapy for these patients. Radiofrequency ablation (RFA), the most common ablation technology, has the advantage of being a more minimally invasive treatment, having a shorter length of hospital stay, and allowing a faster recovery compared with LR (5), which may be associated with better quality of life for HCC patients. Compared with RFA alone, RFA combined with TACE may be more effective because TACE can embolize tumor blood vessels, reduce tumor blood supply, reduce the heat sink effect of hepatic blood flow on thermal coagulation, and may lead to tumor necrosis after tumor hypoxia (6, 7).

However, pairwise comparisons of LR, RFA, and RFA+TACE in many previous studies have shown conflicting results. Comparing LR and RFA, two randomized controlled trials (RCTs) showed longer overall survival (OS) for LR than RFA (8, 9). However, the other three RCTs showed that they were equally effective (10–12), and another RCT did not indicate which one was superior (13). Comparing LR with RFA+TACE, a single RCT showed longer OS and recurrence-free survival (RFS) with LR compared with sequential TACE and RFA (14). However, several observational studies have shown that RFA+TACE has the same long-term effects as LR (15–18). Comparing RFA with RFA+TACE, one RCT showed that there was no statistical difference between the overall survival rates and safety in the two groups (19). However, a recently published single-center RCT showed that combined RFA and TACE was associated with higher long-term survival rates than RFA alone (20).

To compensate for the deficiency of traditional systematic reviews and meta-analyses, we conducted network meta-analysis to integrate the results of direct and indirect comparisons in simultaneously comparing multiple treatment options; the ranking probability and surface under the cumulative ranking curve (SUCRA) index were used to rank the effectiveness of each treatment and to determine which is best based on OS and RFS.

## 2 Material and methods

### 2.1 Protocol and registration

This systematic review including network meta-analysis was conducted based on the guidelines for the PRISMA-NMA extension statements for network meta-analyses (21). We registered our protocol with the PROSPERO International Prospective Register of Systematic Reviews (ID: CRD42022299269, http://www.crd.york.ac.uk/PROSPERO)

### 2.2 Eligibility criteria

The inclusion criteria based on the PICO principle are as follows:

(1) P (patients): The patient is in the very-early- and early-stage of hepatocellular carcinoma (BCLC 0 or A).
(2) I (intervention) and C (comparison): Patient underwent one of the following three interventions: liver resection (LR), radiofrequency ablation (RFA), and radiofrequency ablation combined with transcatheter arterial chemoembolization (RFA+TACE), E.g., LR versus RFA or LR versus RFA+TACE or RFA versus RFA+TACE comparisons.
(3) O (outcomes): Overall survival and recurrence-free survival of combined data will be reported.
(4) S (study): Randomized controlled trials (RCTs) and observational analyses with propensity score-matched cohort analyses (PSMs).

### 2.3 Search strategy

We searched the PubMed, Embase, and Cochrane Library databases from 19 December 2021 to 5 January 2022. There were no restrictions on year of publication or language. To include the latest studies, we conducted a final search of the above databases on 22 March 2022. We searched the databases by combining subject words with free words. The detailed retrieval strategies are included in **Supplementary Table 1**.

### 2.4 Study selection

**Figure 1:**
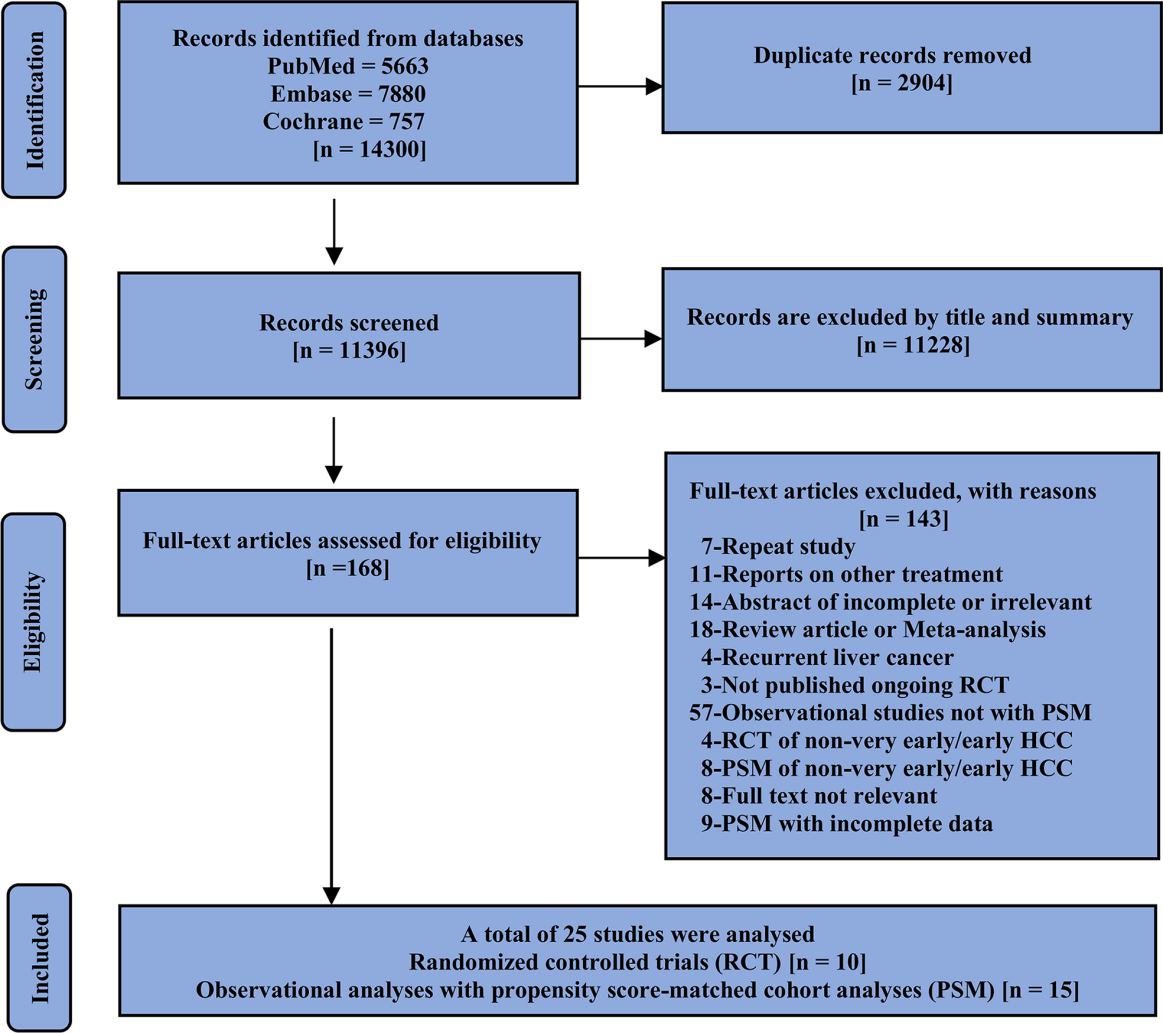
Flow chart showing the selection of studies.

First, duplicate literature from different databases was excluded, and articles were then initially screened by title and abstract followed by a full-text review of the studies of interest; randomized controlled trials (RCTs) and observational analyses with propensity score-matched cohort analyses (PSMs) were also included in our study. The included studies should compare at least two of the three options and report on the outcomes of interest. If the same author or team reported results from the same patient population in multiple journals, our analysis included only the most recent or complete report. We consolidated the data in cases where the short- and long-term results of the same study were presented over different periods. Only studies published as journal articles with complete results were eligible for inclusion in our analysis. The specific screening details and reasons for exclusion are shown in **Figure 1**.

### 2.5 Data extraction and study outcomes

The data were independently extracted into a standardized spreadsheet by the two authors, Zhang and Qin. Any disputes and conflicts were discussed with the third author, Wang, until a consensus was reached. The authors of completed studies were contacted by email as required. We extracted the following data (1): author, year of publication, country, design type of study, several subjects (2); demographic details: age and sex (3); follow-up information: duration of follow-up and equipment used (4); tumor information: size and number, Child–Pugh score, and ECOG (PS) score; and (5) the primary outcomes of this study were 1-, 3-, and 5-year OS, and the secondary outcomes were 1-, 3-, and 5-year RFS. OS and RFS are dichotomous time-to-event data. Therefore, they allow calculating the HR values of both the experimental and control groups for comparison. We extracted the HR values and 95% confidence intervals from all the original studies. If the HR and 95% CI of the OS or RFS were not provided in the original text, we extracted the HR from the Kaplan–Meier curve of the study data. Details of the specific extracted data are in **Supplementary Table 2**.

### 2.6 The geometry of the network

After data extraction was completed, we constructed a network graph with different nodes representing different treatment measures, namely liver resection (LR), radiofrequency ablation (RFA), and radiofrequency ablation combined with transcatheter arterial chemoembolization (RFA+TACE) and evaluated whether the network graph formed a triangle. The circles represent interventions, and the line thickness between the two treatment nodes indicates the number of comparative studies.

### 2.7 Quality assessment

The risk of bias was assessed for all of the included studies. RCTs were evaluated using the Cochrane Risk of Bias 2 tool (ROB 2.0) (22), and observational studies were evaluated using the Robins-I tool (ROBINS-I) (23). We prepared a visual summary of the included study risks according to (24). Detailed information on the risk of bias assessment is provided in **Supplementary Figure 1**.

### 2.8 Statistical analysis

After extracting the available values of the OS and RFS for the included studies, meta-analysis was performed using a generic inverse-variance method; the HR value and its 95% confidence interval were calculated according to the method reported by Woods and Watkins (25, 26). Since there was insufficient evidence to show that the intervention effect sizes of all the included studies were the same, that is, the difference between the observed effect sizes may have been caused by random error and differences between the real intervention effects, we chose a random effects model, although there was no significant heterogeneity in our study. Heterogeneity was explored using subgroup analysis based on the tumor number and diameter. In addition, we also carried out inconsistency assessment and network meta-regression. Direct meta-analysis of the pair comparisons was performed using Review Manager Software version 5.4. Bayesian random effects network meta-analysis was performed using R version 4.1.2 (https://cran.r-project.org). The “gemtc” and “rjags” packages were used for analysis. For the MCMC simulation, the “gemtc” package communicates with the JAGS program in the background *via* the “rjags” package.

## 3 Results

### 3.1 Included studies

A total of 25 studies (10 RCTs and 15 PSMs) fulfilled the inclusion criteria and were included in the network meta-analysis. The studies included in this analysis were published between 2006 and 2021; fifteen studies were conducted in China, six in Korea, three in Japan, and one in Italy. In total, 4249 patients were pooled for the meta-analysis. Six RCTs (960 patients) and eleven PSMs (2252 patients) compared LR with RFA (8–13, 27–37). One RCT (200 patients) and four PSMs (522 patients) compared LR with RFA+TACE (14–18). Only three RCTs (315 patients) compared RFA+TACE with RFA (19, 20, 38). Among these, 1952 patients were under the LR arm, 1814 patients were under the RFA arm, and 483 patients were under the RFA+TACE arm. In these studies, with the exception of Ye et al. ‘s study (31) that did not report specific age information or follow-up time, the age (median or mean) range of the other included individuals was 47.0–73.0, and the follow-up time (months) range was 24.2–93.0. Almost all of the participants had good liver function, with Child–Pugh class A or B. The size and number of tumors were consistent with very-early- and early-stage HCC (BCLC 0 or A). Details of the selected studies are shown in **Table 1**.

**Table 1:** A list of all included studies.

Study	Type	Year andcountry	Arm	No. ofpatients	Age (mean or median)	Sex(M/F)	Child–Pugh	HBV/HCV	Mean tumorsize(cm)	Tumornumber
Li (27)	PSM	China, 2021	LR vs. RFA	58 vs. 58	61.0 vs. 61.0	39/19 vs. 39/19	A vs. A	28/34 vs. 23/27	1.90 vs. 1.80	1
Lee (28)	PSM	Korea, 2021	LR vs. RFA	118 vs. 118	59.5 vs. 60.5	91/27 vs. 88/30	A vs. A	90/10 vs. 84/12	1.84 vs. 1.87	1
Pan (29)	PSM	China, 2020	LR vs. RFA	118 vs. 236	53.0 vs. 56.0	101/17 vs. 206/30	A, B vs. A, B	NA	2.50 vs. 2.55	≤3
Chong (30)	PSM	China, 2020	LR vs. RFA	59 vs. 59	57.7 vs. 59.3	46/13 vs. 46/13	A vs. A, B	48/4 vs. 48/4	2.00 vs. 2.30	≤3
Ye (31)	PSM	China, 2019	LR vs. RFA	154 vs. 154	NA	141/13 vs. 134/20	A, B vs. A, B	135/2 vs. 134/5	3-5 vs. 3-5	1
Kim. T. H (32).	PSM	Korea, 2019	LR vs. RFA	48 vs. 48	56.2 vs. 58.7	38/10 vs. 35/13	A vs. A	36/5 vs. 34/8	1.57 vs. 1.53	1
Lee. H. W (8).	RCT	Korea, 2018	LR vs. RFA	29 vs. 34	55.6 vs. 56.1	23/6 vs. 24/10	A vs. A	NA	2-4 vs. 2-4	1
Ng. K.K.C (13).	RCT	China, 2017	LR vs. RFA	109 vs. 109	55.0 vs. 57.0	89/20 vs. 86/23	A, B vs. A, B	99/5 vs. 95/0	2.90 vs. 2.60	≤3
Song (33)	PSM	China, 2016	LR vs. RFA	78 vs. 78	48.0 vs. 48.0	70/8 vs. 70/8	A vs. A, B	73/NA vs. 77/NA	<4 vs.<4	1
Liu (34)	PSM	China, 2016	LR vs. RFA	79 vs. 79	61.0 vs. 63.0	55/24 vs. 52/27	A vs. A	46/31 vs. 36/30	≤2 vs. ≤2	1
Kang. T. W (35).	PSM	Korea, 2015	LR vs. RFA	99 vs. 99	54.0 vs. 55.0	77/22 vs. 77/22	A, B vs. A, B	83/8 vs. 83/8	2.00 vs. 1.90	1
Jiang (36)	PSM	China, 2015	LR vs. RFA	140 vs. 140	53.0 vs. 55.0	123/17 vs. 118/22	A, B vs. A, B	129/NA vs. 121/NA	2.40 vs. 2.30	≤3
Fang (10)	RCT	China, 2014	LR vs. RFA	60 vs. 60	53.5 vs. 51.4	46/14 vs. 42/18	A, B vs. A, B, C	52/NA vs. 55/NA	2.28 vs. 2.21	≤3
Pompili (37)	PSM	Italy, 2013	LR vs. RFA	116 vs. 116	67.0 vs. 69.0	87/29 vs. 92/24	A vs. A	11/78 vs. 17/78	2.30 vs. 2.30	1
Feng (11)	RCT	China, 2012	LR vs. RFA	84 vs. 84	47.0 vs. 51.0	75/9 vs. 79/5	A, B vs. A, B	NA	2.60 vs. 2.40	≤2
Huang (9)	RCT	China, 2010	LR vs. RFA	115 vs. 115	55.9 vs. 56.6	85/30 vs. 79/36	A, B vs. A, B	104/6 vs. 101/4	≤5 vs. ≤5	≤3
Chen (12)	RCT	China, 2006	LR vs. RFA	90 vs. 71	49.4 vs. 51.9	75/15 vs. 56/15	A vs. A	NA	≤5 vs. ≤5	1
Lee. H. J (15).	PSM	Korea, 2019	LR vs. RFA+TACE	26 vs. 26	59.6 vs. 62.4	22/4 vs. 21/5	A vs. A, B	13/7 vs. 14/6	3.58 vs. 3.60	1
Lee. H. J (16)	PSM	Korea, 2017	LR vs. RFA+TACE	49 vs. 49	60.8 vs. 61.7	37/12 vs. 37/12	A vs. A	33/7 vs. 36/3	2.47 vs. 2.55	1
Bholee (17)	PSM	China, 2017	LR vs. RFA+TACE	148 vs. 74	52.2 vs. 54.9	136/12 vs. 68/6	A, B vs. A, B	135/2 vs. 70/4	3.00 vs. 2.90	≤3
H. Liu (14)	RCT	China, 2016	LR vs. RFA+TACE	100 vs. 100	49.0 vs. 52.0	94/6 vs. 86/14	A, B vs. A, B	90/NA vs. 87/NA	3.00 vs. 2.80	≤3
Takuma (18)	PSM	Japan, 2013	LR vs. RFA+TACE	75 vs. 75	70.0 vs. 70.0	48/27 vs. 56/19	A, B vs. A, B	5/60 vs. 4/62	2.30 vs. 2.20	≤3
Zhang (20)	RCT	China, 2021	RFA vs. RFA+TACE	95 vs. 94	55.3 vs. 53.3	71/24 vs. 75/19	A, B vs. A, B	83/6 vs. 85/6	3.39 vs. 3.47	≤3
Morimoto (19)	RCT	Japan, 2010	RFA vs. RFA+TACE	18 vs. 19	73.0 vs. 70.0	12/6 vs. 15/4	A, B vs. A, B	0/16 vs. 0/17	3.70 vs. 3.60	1
Shibata (38)	RCT	Japan, 2009	RFA vs. RFA+TACE	43 vs. 46	69.8 vs. 67.2	33/10 vs. 31/15	A, B vs. A, B	9/30 vs. 12/32	1.60 vs. 1.70	≤3

RCT, randomized controlled trial; PSM, propensity score-matched cohort analysis; M, male; F, female; HBV, hepatitis B viral infection; HCV, hepatitis C viral infection; LR, liver resection; RFA, radiofrequency ablation; TACE, transcatheter arterial chemoembolization; NA, not available.

### 3.2 Risk of bias within studies

We assessed the risk of bias for each of the studies, weighted according to the sample size of the study (**Supplementary Figure 1**). In general, although the studies we included all met the pre-established PICOS criteria, they all demonstrated a certain publication bias. The ROB2 tool was used to evaluate the 10 RCTs included in the meta-analysis. Five RCTs were evaluated as having a general high-risk bias. The reports of Fang et al. (10), Chen et al. (12), and Shibata et al. (38) did not correspond to a completely randomized design. A risk of deviation from the intended intervention was identified for the study by Lee. H. W et al. (8). The study by Morimoto et al. (19) had an absence of data categories. Therefore, these studies were considered to have an overall high risk of bias. The Robins-I tool was used to evaluate 15 NRCTs included in the meta-analysis. Six NRCTs were assessed as having an overall serious risk. The studies of Pan et al. (29) and Bholee et al. (17) were found to be biased due to confounding factors. The study by Chong et al. (30) may have had a bias in the selection of participants; that by Kim. T. H et al. (32) showed deviation from the expected intervention; and that by Lee. H. J et al. (15, 16) had missing data. Thus, these studies were classified as having a serious risk. Due to the nature of the intervention, all the studies using double-blind techniques were considered inadequate in terms of blinding.

### 3.3 Results of the pairwise meta-analysis

#### 3.3.1 LR vs. RFA

Regarding OS, LR was not significantly different from RFA in the short term (at one year, HR: 0.93, 95% CI: 0.59-1.47). However, LR showed a superior effect to that of RFA in terms of long-term comparison (at three years, HR: 0.75, 95% CI: 0.58-0.97; at five years, HR: 0.71, 95% CI: 0.55-0.92). In RFS, compared with RFA, LR showed superior efficacy in both long-term and short-term outcomes (at one year, HR: 0.66, 95% CI: 0.51-0.84; at three years, HR: 0.69, 95% CI: 0.58-0.82; at five years, HR: 0.61, 95% CI: 0.48-0.78). The specific data analysis is shown in **Supplementary Figure 2**.

#### 3.3.2 LR vs. RFA+TACE

Regarding short- and long-term OS results, there was no significant difference between LR and RFA+TACE (at one year, HR: 1.52, 95% CI: 0.72-3.21; at three years, HR: 0.91, 95% CI: 0.51-1.62; at five years, HR: 0.95, 95% CI: 0.64-1.42). Regarding RFS, similar to OS, LR does not seem to show an advantage over RFA+TACE (at one year, HR: 1.45, 95% CI: 0.59-3.55; at three years, HR: 0.82, 95% CI: 0.54-1.24; at five years, HR: 0.83, 95% CI: 0.65-1.06). The specific data analysis is shown in **Supplementary Figure 3**.

#### 3.3.3 RFA+TACE vs. RFA

Regarding OS, RFA+TACE appears to be superior to RFA in the short term (at one year, HR: 0.33, 95% CI: 0.12-0.89). However, there was no significant difference in the long-term outcomes between the two treatments (at three years, HR: 0.70, 95% CI: 0.45-1.07; at five years, HR: 0.80, 95% CI: 0.55-1.19). Regarding RFS, compared with RFA, RFA+TACE showed similar treatment effects in the short-term outcomes (at one year, HR: 0.74, 95% CI: 0.36-1.52). However, in the long term, RFA+TACE showed better therapeutic effects (at three years, HR: 0.62, 95% CI: 0.44-0.86; at five years, HR: 0.68, 95% CI: 0.47-0.99). The specific data analysis is shown in **Supplementary Figure 4**.

### 3.4 Results of the network meta-analysis

The network graph comparing the three treatments formed a complete triangle (**Supplementary Figure 5**), with the largest number of studies comparing LR with RFA (OS at one year: 14; OS at three years: 16; OS at five years: 10; RFS at one year: 14; RFS at three years: 16; RFS at five years: 10), followed by LR with RFA+TACE (OS at one year: 5; OS at three years: 5; OS at five years: 5; RFS at one year: 5; RFS at three years: 5; RFS at five years: 5). RFA+TACE had the lowest number of studies compared to RFA (OS at one year: 3; OS at three years: 3; OS at five years: 2; RFS at one year: 2; RFS at three years: 2; RFS at five years: 1).

**Figure 2:**
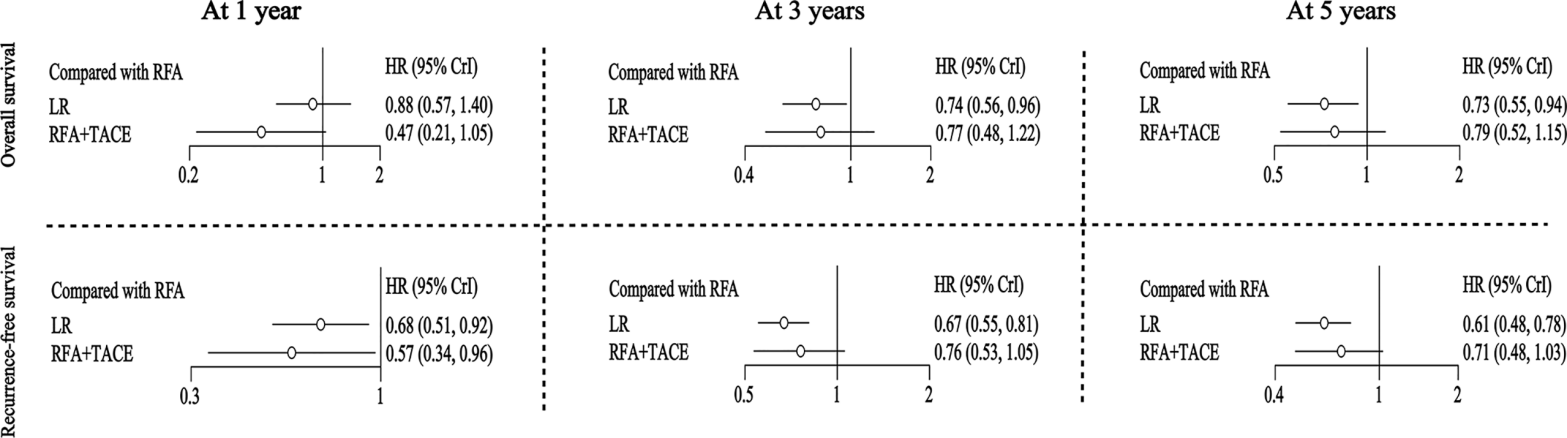
Hazard ratios (for OS and RFS) along with 95% confidence intervals (CIs) for LR and RFA+TACE compared with RFA for all the included studies.

For the outcome of OS at one year, LR had an HR of 0.88 (95% CI: 0.57-1.40), and RFA+TACE had an HR of 0.47 (95% CI: 0.21-1.05) compared with RFA. For the OS at three years, LR had an HR of 0.74 (95% CI: 0.56-0.96), and RFA+TACE had an HR of 0.77 (95% CI: 0.48-1.22) compared with RFA. For the OS at five years, LR had an HR of 0.73 (95% CI: 0.55-0.94), and RFA+TACE had an HR of 0.79 (95% CI: 0.52-1.15) compared with RFA. Overall, LR was found to be associated with higher rates of long-term OS than RFA. There was no significant difference in OS between RFA+TACE and RFA alone (**Figure 2** and **Table 2**).

**Table 2:** Summary of all results.

	Treatment	LR	RFA	RFA+TACE
OS at 1 year	LR vs.	NA	0.88 (0.57,1.40)	1.87 (0.89,3.96)
	RFA vs.	1.13 (0.71,1.75)	NA	2.12 (0.95,4.67)
	RFA+TACE vs.	0.53 (0.25,1.12)	0.47 (0.21,1.05)	NA
OS at 3 years	LR vs.	NA	0.74 (0.56,0.96)	0.96 (0.61,1.49)
	RFA vs.	1.35 (1.04,1.80)	NA	1.30 (0.82,2.10)
	RFA+TACE vs.	1.04 (0.67,1.63)	0.77 (0.48,1.22)	NA
OS at 5 years	LR vs.	NA	0.73 (0.55,0.94)	0.92 (0.66,1.31)
	RFA vs.	1.37 (1.07,1.81)	NA	1.27 (0.87,1.91)
	RFA+TACE vs.	1.08 (0.76,1.52)	0.79 (0.52,1.15)	NA
RFS at 1 year	LR vs.	NA	0.68 (0.51,0.92)	1.20 (0.73,2.00)
	RFA vs.	1.46 (1.08,1.98)	NA	1.76 (1.04,2.98)
	RFA+TACE vs.	0.83 (0.50,1.38)	0.57 (0.34,0.96)	NA
RFS at 3 years	LR vs.	NA	0.67 (0.55,0.81)	0.88 (0.65,1.22)
	RFA vs.	1.49 (1.24,1.81)	NA	1.32 (0.95,1.87)
	RFA+TACE vs.	1.13 (0.82,1.54)	0.76 (0.53,1.05)	NA
RFS at 5 years	LR vs.	NA	0.61 (0.48,0.78)	0.86 (0.62,1.21)
	RFA vs.	1.63 (1.29,2.09)	NA	1.40 (0.97,2.09)
	RFA+TACE vs.	1.16 (0.82,1.60)	0.71 (0.48,1.03)	NA

OS, overall survival; RFS, recurrence-free survival; LR, liver resection; RFA, radiofrequency ablation; TACE, transcatheter arterial chemoembolization; NA, not available.

For the outcome of RFS at one year, LR and RFA+TACE had an HR of 0.68 (95% CI: 0.51-0.92) and 0.57 (95% CI: 0.34-0.96), respectively, compared with RFA. For the outcome of RFS at three years, LR and RFA+TACE had an HR of 0.67 (95% CI: 0.55-0.81) and 0.76 (95% CI: 0.53-1.05), respectively, compared with RFA. For the outcome of RFS at five years, LR and RFA+TACE had an HR of 0.61 (95% CI: 0.48-0.78) and 0.71 (95% CI: 0.48-1.03), respectively, compared with RFA. Overall, LR outperformed RFA in terms of overall RFS. RFA+TACE was found to have higher rates of one-year RFS than RFA; however, there may be no significant difference between the three-year and five-year RFS (**Figure 2** and **Table 2**).

### 3.5 Treatment rankings and probability

**Figure 3:**
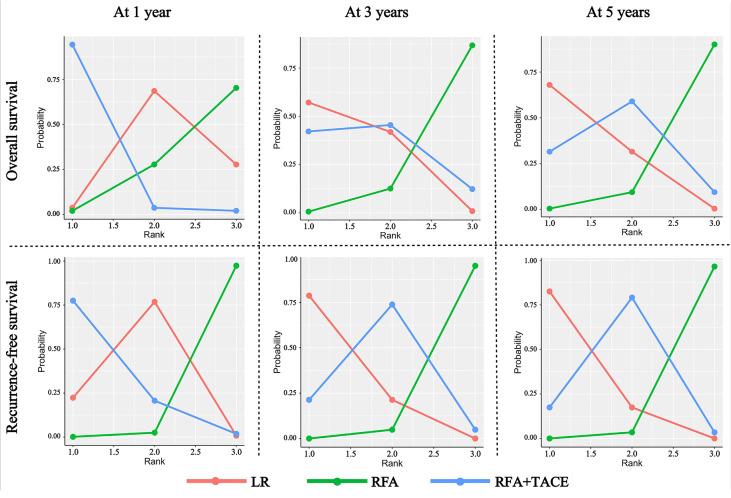
Rank probability of LR, RFA+TACE, and RFA based on each outcome criteria evaluated in the study.

Under the Bayesian framework, we calculated the rank probability of all the treatment comparisons, and the higher the probability under each rank, the higher the likelihood that the particular treatment would be the optimal intervention (**Figure 3**); the SUCRA values are presented in **Supplementary Figure 6**. For OS at one year, RFA+TACE was found to have the highest probability of ranking first, LR has the highest probability of ranking second, and RFA alone has the highest probability of ranking third. For OS at three years, LR, RFA+TACE, and RFA alone were found to have the highest probability of ranking from 1 to 3, respectively. The results of the OS ranking at five years are similar to those of the OS ranking at three years. For RFS at one year, RFA+TACE was found to have the highest probability of ranking first, LR has the highest probability of ranking second, and RFA alone has the highest probability of ranking third. For RFS at three years, LR, RFA+TACE, and RFA alone were found to have the highest probability of ranking from 1 to 3, respectively. The results of the RFS ranking at five years are similar to those of the RFS ranking at three years. For the SUCRA values, RFA+TACE ranked the highest in cumulative value for short-term outcomes, and LR ranked the highest in the long-term outcomes for both OS and RFS. The specific values of the above results are given in **Supplementary Table 3**.

### 3.6 Network consistency and publication bias

The analysis model needed to be set up before perform Bayesian operations using the “gemtc” package. We set specific commands as follows: type=consistency, n.chain=4, likelihood=binom, link=cloglog, linearModel=random; after the model was established, JAGS was invoked using gemtc for MCMC iterative calculation. The number of adaptive iterations was kept at 20000, and the number of simulation iterations was kept at 50000. The degree of convergence of all the operations related to the consistency model was satisfactory; the median value of the shrink factor and 97.5% of the shrink factor tended to approach 1 and reached stability after the iterative calculation; the PSRF (potential scale reduction factor) value tended to approach 1. The diagnostic results of the developed model are shown in **Supplementary Table 4**. For the meta-analysis of paired comparisons, the publication bias was presented in the form of a funnel plot, shown in **Supplementary Figure 7**. For the comparative analysis with a number greater than or equal to 10, the results are relatively symmetric. As shown in **Supplementary Table 4**, the overall I^2^ values of all the networks that made up the mesh analysis were less than 20%. In general, each subset of this study had good consistency and low heterogeneity.

### 3.7 Analysis of inconsistency

The inconsistencies of all the closed-loop structures were detected using the node-splitting method, and none of the P-values from comparison indicated significant inconsistency. Therefore, direct and indirect comparisons demonstrated good consistency (**Supplementary Figure 8**).

### 3.8 Subgroup analysis

#### 3.8.1 According to the number of tumors

We performed a subgroup analysis of studies that included only single tumors, all of which were less than 5cm in diameter (8, 12, 15, 16, 19, 27, 28, 31–35, 37). For the outcome of OS at one year, LR had an HR of 1.12 (95% CI: 0.52-2.21), and RFA+TACE had an HR of 0.50 (95% CI: 0.07-3.11) compared with RFA. For the OS at three years, LR had an HR of 0.82 (95% CI: 0.56-1.14), and RFA+TACE had an HR of 0.74 (95% CI: 0.26-1.92) compared with RFA. For the OS at five years, LR had an HR of 0.78 (95% CI: 0.55-1.12), and RFA+TACE had an HR of 0.68 (95% CI: 0.34-1.39) compared with RFA. Overall, the three treatments did not demonstrate statistically significant differences (**Supplementary Table 5** and **Supplementary Figure 9**).

For the outcome of RFS at one year, LR and RFA+TACE had an HR of 0.65 (95% CI: 0.39-1.06) and 0.26 (95% CI: 0.05-1.16), respectively, compared with RFA. For the outcome of RFS at three years, LR and RFA+TACE had an HR of 0.70 (95% CI: 0.53-0.90) and 0.52 (95% CI: 0.24-1.08), respectively, compared with RFA. For the outcome of RFS at five years; LR and RFA+TACE had an HR of 0.68 (95% CI: 0.49-0.93) and 0.54 (95% CI: 0.27-1.08), respectively, compared with RFA. Overall, the three- and five-year RFS of LR were superior to those of RFA. The combination of RFA and TACE did not show statistically significant differences at one, three, and five years of RFS compared with RFA alone. (**Supplementary Table 5** and **Supplementary Figure 9**). We ranked the comparisons in the subgroup analysis and analyzed the inconsistencies of the comparisons in the closed-loop network using the node-splitting method (**Supplementary Table 6** and **Supplementary Figure 10**).

#### 3.8.2 Based on tumor diameter

Subgroup analyses were also performed according to tumor diameter, with the mean tumor diameter of all included studies being less than or equal to 3 cm. For the outcome of OS at one year, LR had an HR of 0.97 (95% CI: 0.56-1.77), and RFA+TACE had an HR of 0.66 (95% CI: 0.24-1.90) compared with RFA. For the OS at three years, LR had an HR of 0.81 (95% CI: 0.56-1.14), and RFA+TACE had an HR of 0.89 (95% CI: 0.47-1.63) compared with RFA. For the OS at five years, LR had an HR of 0.71 (95% CI: 0.46-1.07), and RFA+TACE had an HR of 0.73 (95% CI: 0.34-1.39) compared with RFA. Overall, the three treatments did not demonstrate statistically significant differences (**Supplementary Table 7** and **Supplementary Figure 11**).

For the outcome of RFS at one year, LR and RFA+TACE had an HR of 0.66 (95% CI: 0.42-1.06) and 0.59 (95% CI: 0.28-1.30), respectively, compared with RFA. For the outcome of RFS at three years, LR and RFA+TACE had an HR of 0.66 (95% CI: 0.51-0.85) and 0.85 (95% CI: 0.54-1.32), respectively, compared with RFA. For the outcome of RFS at five years; LR and RFA+TACE had an HR of 0.59 (95% CI: 0.39-0.86) and 0.72 (95% CI: 0.37-1.35), respectively, compared with RFA. Overall, the three- and five-year RFS of LR were superior to those of RFA. The combination of RFA and TACE did not show statistically significant differences at one, three, and five years of RFS compared with RFA alone. (**Supplementary Table 7** and **Supplementary Figure 11**). We ranked the comparisons in the subgroup analysis and analyzed the inconsistencies of the comparisons in the closed-loop network using the node-splitting method (**Supplementary Table 8** and **Supplementary Figure 12**).

### 3.9 Network meta-regression

Using a regression model, three covariables (study type, publication year, and sample size) were discussed, respectively, to determine whether these had an impact on the results. **Supplementary Table 9** shows the shared beta coefficients and the 95% confidence intervals for the three regression parameters. The confidence intervals of the six comparative beta coefficients of the three covariables all contain 0. Therefore, it can be considered that the outcome did not significantly differ for any of the three covariables; that is, the studied covariables did not influence the treatment effect.

## 4 Discussions

The main objective of our study was to compare the efficacy of the three treatments for patients with BCLC 0/A hepatocellular carcinoma. Some previous pairwise meta-analyses compared OS and RFS at one, three, and five years using OR or RR but ignored the concept of time. However, OS and RFS are time-to-event variables, so consideration of HR probably allows for the most accurate comparisons. Although the results of the network meta-analysis indicated there were no differences in OS and RFS between LR and RFA+TACE across all comparisons, the probability ranking showed a higher probability of RFA+TACE ranking first for short-term outcomes (at one year), whereas for long-term outcomes (at three and five years), the likelihood of LR ranking first was significantly higher. The results of direct comparison and network comparison between RFA+TACE versus RFA alone may seem inconsistent in terms of RFS outcomes at one, three, and five years, but the small sample size (only 37 patients) included in Morimoto et al.’s study (19) and there being only one study by Zhang et al. (20) for the comparison of outcomes at year 5 reduced the reliability of the direct comparison; therefore, the results of the network meta-analysis may be more accurate.

In recent years, regional therapies, including TACE (with or without chemotherapeutic) and RFA, have become key components of hepatocellular carcinoma treatment. Previous studies have shown no significant difference between RFA and LR regarding OS for single tumors smaller than 3 cm (10–12); however, LR may still result in better outcomes than RFA when it comes to RFS (39). Rapid advances in radiofrequency ablation equipment, improved electrodes, and the widespread use of high-resolution intraoperative ultrasound have increased the advantages of RFA. Although RFA is superior to LR in terms of being minimally invasive and its safety, its use has some unavoidable limitations, i.e., tumor location is an important determinant of the choice of ablation approach, especially when the tumor is in the gallbladder, gastrointestinal tract, bile duct, or near the diaphragm; percutaneous radiofrequency ablation may cause thermal damage to adjacent organs; thus, considering a laparoscopic approach at this time or open operation for RFA may be necessary. RFA combined with TACE is a common method for the clinical treatment of HCC. Combined therapy can produce a synergistic effect; TACE can reduce the formation of heat sinks during ablation and increase the ablation area, and thermal ablation can increase the efficacy of chemotherapeutic drugs. Previous studies have shown that sequential therapy with TACE and RFA plays a clear role in the recovery of patients with intermediate and advanced HCC (40, 41). For very-early- and early-stage HCC, our study showed that RFA+TACE was superior to RFA alone in terms of short-term RFS (at one year), but there was no significant difference between the two treatments in terms of OS at one, three, and five years. The number of tumors and the tumor diameter is vital for the choice of clinical treatment, and the Barcelona staging system based on the number and diameter of tumor recommended different treatments, we had a subgroup analysis, based on a group for those who are single tumor research, another group for the research of tumor diameter less than or equal to 3 cm. Numerous previous studies have shown that ablation is as effective as a surgical resection for tumors smaller than 3cm; however, whether ablation combined with TACE provides better results remains unknown. The results of subgroup analysis showed that there was no statistically significant difference in tumor number and tumor diameter between combined therapy and RFA alone. Therefore, the advantages of combination therapy in patients with very-early-and early-stage HCC are limited. Recent studies have shown that incomplete embolization and lipiodol deposition after TACE in combination therapy may result in the omission of lesions and affect the efficacy, increasing the risk of adverse postoperative events (42, 43). In addition, the time interval between TACE and RFA remains controversial for sequential therapy, so the efficacy of RFA+TACE combination therapy remains uncertain.

Additionally, due to the widespread use of laparoscopy and liver segment or liver lobe staining, traditional hepatectomy has been greatly improved in terms of its safety, minimal invasiveness, and effectiveness. This has led to a dramatic increase in liver cancer patients undergoing laparoscopic and robotic surgery. Laparoscopic hepatectomy (LH) is considered safe and effective treatment for liver cancer. Studies have shown that LH has almost the same effect as traditional open surgery on HCC patients in terms of OS and RFS (44, 45); moreover, it is associated with a lower complication rate, less intraoperative blood loss, and shorter hospital stay (46, 47). Several recent studies comparing laparoscopic hepatectomy with percutaneous radiofrequency ablation have shown that LH is superior to percutaneous radiofrequency ablation for very-early- or early-stage HCC (28, 30, 48). In addition, considering tumor location, LH is superior to percutaneous radiofrequency ablation for small, single HCC tumors located in subcapsular, perivascular, and anteromedial extrahepatic segments (28). However, there is currently no evidence that LH is comparable with RFA in terms of complication rates and length of hospital stay, so further studies are needed to clarify this.

Our study also has some limitations. First, we combined the results of randomized controlled trials with those from observational studies. Although the observational studies included propensity score matching, they may have some deviations compared with randomized controlled trials. We cannot judge the quality of the direct and indirect comparisons based on the method of GRADE scoring. Second, although we conducted regression analysis on the publication year, the overall study period between LR and RFA was relatively long. With the continuous upgrading of ablation technology, new technologies may have advantages over previous ones, and the influence of this has not been determined. Third, the data of all included studies were generally sparse regarding liver function status, tumor location, choice of radiofrequency ablation electrodes, and complications. However, effective analysis of these confounding factors will be crucial for the treatment choice of patients.

Although our study has some limitations, our results have some significance for guiding clinical practice. In summary, this systematic review and meta-analysis showed that LR is superior to RFA alone in terms of its overall efficacy, regardless of whether the OS or RFS are considered. The combination of RFA+TACE was superior to RFA alone only at one-year RFS, while there were no significant differences at one, three, and five years of OS, and at three and five years of RFS.

## Data availability statement

The original contributions presented in the study are included in the article/**Supplementary Material**. Further inquiries can be directed to the corresponding author.

## Author contributions

All authors contributed to the article and approved the submitted version.

## Funding

This work was supported in part by grants from the National Natural Science Foundation of China (No.81501562); the Shandong Province Science and Technology Development Project (No.2019WS596); and the Shandong Provincial Natural Science Foundation (No. ZR2020MH293).

## Conflict of interest

The authors declare that the research was conducted in the absence of any commercial or financial relationships that could be construed as a potential conflict of interest.

## Publisher’s note

All claims expressed in this article are solely those of the authors and do not necessarily represent those of their affiliated organizations, or those of the publisher, the editors and the reviewers. Any product that may be evaluated in this article, or claim that may be made by its manufacturer, is not guaranteed or endorsed by the publisher.
