# Supplementary material for: Liver resection, radiofrequency ablation, and radiofrequency ablation combined with transcatheter arterial chemoembolization for very-early- and early-stage hepatocellular carcinoma: A systematic review and Bayesian network meta-analysis for comparison of efficacy
Source: Front Oncol. 2022 Oct 28;12:991944. doi: 10.3389/fonc.2022.991944 (PMC9650158; doi:10.3389/fonc.2022.991944)

Supplementary Material

**Supplementary Table 1.** Detailed search strategy.

**Supplementary Table 2.** Details of various studies included in meta-analysis

**Supplementary Table 3.** Rank probability table for all studies.

**Supplementary Table 4.** Model diagnostics of the developed consistency model for the outcomes of the study.

**Supplementary Table 5.** League Table of subgroup analysis (Single tumor studies).

**Supplementary Table 6.** Rank probability table for subgroup analysis (Single tumor studies).

**Supplementary Table 7.** League Table of subgroup analysis (Tumor diameter ≤ 3cm).

**Supplementary Table 8.** Rank probability table for subgroup analysis (Tumor diameter ≤ 3cm).

**Supplementary Table 9.** The shared beta coefficients and 95% confidence intervals for the three regression parameters.

**Supplementary Figure 1.** Quality assessment for all studies.

**Supplementary Figure 2.** Pairwise meta-analysis: LR vs RFA.

**Supplementary Figure 3.** Pairwise meta-analysis: LR vs RFA+TACE.

**Supplementary Figure 4.** Pairwise meta-analysis: RFA+TACE vs RFA.

**Supplementary Figure 5.** Graph of network nodes included in the study.

**Supplementary Figure 6.** SUCRA plot for all results.

**Supplementary Figure 7.** To assess publication bias, funnel plots were made for Pairwise meta-analyses involving more than 10 studies.

**Supplementary Figure 8.** The inconsistencies of all closed-loop structures are detected by the Node-Splitting method. (All studies)

**Supplement Figure 9.** Hazard ratio (for OS and RFS) along with 95% credible interval (CI) for LR and RFA+TACE compared with RFA for the subgroup analysis (Single tumor studies).

**Supplementary Figure 10.** The inconsistencies of all closed-loop structures are detected by the Node-Splitting method. (Subgroup analysis for the Single tumor studies)

**Supplement Figure 11.** Hazard ratio (for OS and RFS) along with 95% credible interval (CI) for LR and RFA+TACE compared with RFA for the subgroup analysis (Tumor diameter ≤ 3cm).

**Supplementary Figure 12.** The inconsistencies of all closed-loop structures are detected by the Node-Splitting method. (Subgroup analysis for the tumor diameter ≤ 3cm)

| **Supplementary Table 1.** Detailed search strategy. | |
| --- | --- |
| PUBMED | |
| #1 | "Hepatectomy"[MeSH Terms] OR "surgical procedures, operative"[MeSH Terms] OR "Microsurgery"[MeSH Terms] OR "Hepatectomy"[Title/Abstract] OR "Hepatectomies"[Title/Abstract] OR "hepatic resection"[Title/Abstract] OR "surgical resection"[Title/Abstract] OR "liver surgery"[Title/Abstract] OR "liver resection"[Title/Abstract] OR "Resection"[Title/Abstract] OR "surgery"[Title/Abstract] |
| #2 | "Radiofrequency Ablation"[MeSH Terms] OR "Radiofrequency Ablation"[Title/Abstract] OR "radio frequency ablation"[Title/Abstract] OR "Ablation"[Title/Abstract] OR "Radiofrequency"[Title/Abstract] OR "rfa therapy"[Title/Abstract] OR "thermal ablation"[Title/Abstract] OR "intervention ablation"[Title/Abstract] OR "interventional ablation"[Title/Abstract] OR "radiological ablation"[Title/Abstract] OR "RFA"[Title/Abstract] |
| #3 | "chemoembolization, therapeutic"[MeSH Terms] OR ("transcatheter arterial chemoembolization"[Title/Abstract] OR "Embolization"[Title/Abstract] OR "transarterial chemoembolization"[Title/Abstract] OR "transarterial embolization"[Title/Abstract] OR "arterial chemoembolisation"[Title/Abstract] OR "arterial chemoembolization"[Title/Abstract] OR "chemoembolisation"[Title/Abstract] OR "transarterial chemoembolisation"[Title/Abstract] OR "transcatheter arterial chemoembolisation"[Title/Abstract] OR ("Transcatheter"[All Fields] AND "chemoembolisation"[Title/Abstract]) OR "transcatheter chemoembolization"[Title/Abstract] OR ("Transcatheter"[All Fields] AND "oily chemoembolisation"[Title/Abstract]) OR "transcatheter oily chemoembolization"[Title/Abstract]) OR "TACE"[Title/Abstract] |
| #4 | "carcinoma, hepatocellular"[MeSH Terms] OR "hepatocellular carcinomas"[Title/Abstract] OR "hepatocellular carcinoma"[Title/Abstract] OR "liver cell carcinoma"[Title/Abstract] OR "liver cell carcinomas"[Title/Abstract] OR "hepatic cancer"[Title/Abstract] OR "hepatocellular cancer"[Title/Abstract] OR "hepatic malignancy"[Title/Abstract] OR "hepatic tumor"[Title/Abstract] OR "liver cancer"[Title/Abstract] OR "liver carcinoma"[Title/Abstract] OR "liver malignancy"[Title/Abstract] OR "liver tumor"[Title/Abstract] OR "hepatocellular malignancy"[Title/Abstract] OR "cancer of liver"[Title/Abstract] OR "malignancy of liver"[Title/Abstract] OR "carcinoma of liver"[Title/Abstract] OR "hepatic neoplasm"[Title/Abstract] OR "neoplasm of liver"[Title/Abstract] OR "hepatocellular neoplasm"[Title/Abstract] OR "hepatocellular neoplasia"[Title/Abstract] OR "liver neoplasia"[Title/Abstract] OR "hepatic neoplasia"[Title/Abstract] OR "Hepatoma"[Title/Abstract] OR "Hepatomas"[Title/Abstract] OR "HCC"[Title/Abstract] |
| #5 | (#1 AND #2 OR (#1 AND #3) OR (#2 AND #3) OR (#1 AND #2 AND #3)) AND #4 |
| #6 | "Randomized Controlled Trial"[Publication Type] OR "Controlled Clinical Trial"[Publication Type] OR "Random Allocation"[MeSH Terms] OR "Comparative Study"[Publication Type] OR "Case-Control Studies"[MeSH Terms] OR "Cohort Studies"[MeSH Terms] OR "Randomized Controlled Trial"[All Fields] OR "randomised controlled study"[All Fields] OR "randomised controlled trial"[All Fields] OR "randomized controlled study"[All Fields] OR "Controlled Clinical Trial"[All Fields] OR "randomized trial"[All Fields] OR "random*"[All Fields] OR "control*"[All Fields] OR "compared"[All Fields] OR "groups"[All Fields] OR "cohort"[All Fields] OR "case control"[All Fields] OR "multivariate"[All Fields] OR "case control*"[All Fields] OR "propensity score"[All Fields] OR "matched"[All Fields] OR "cohort analyses"[All Fields] |
| #7 | #5 AND #6 |
| EMBASE | |
| #1 | 'liver resection'/exp OR hepatectomy:ti,ab,kw OR hepatectomies:ti,ab,kw OR 'hepatic resection':ti,ab,kw OR 'surgical resection':ti,ab,kw OR 'liver surgery':ti,ab,kw OR 'liver resection':ti,ab,kw OR resection:ti,ab,kw OR surgery:ti,ab,kw |
| #2 | 'radiofrequency ablation'/exp OR 'radiofrequency ablation':ti,ab,kw OR 'radio frequency ablation':ti,ab,kw OR ablation:ti,ab,kw OR radiofrequency:ti,ab,kw OR 'rfa therapy':ti,ab,kw OR 'thermal ablation':ti,ab,kw OR 'intervention ablation':ti,ab,kw OR 'interventional ablation':ti,ab,kw OR 'radiological ablation':ti,ab,kw OR rfa:ti,ab,kw |
| #3 | 'chemoembolization'/exp OR 'transcatheter arterial chemoembolization':ti,ab,kw OR embolization:ti,ab,kw OR 'transarterial chemoembolization':ti,ab,kw OR 'transarterial embolization':ti,ab,kw OR 'arterial chemoembolisation':ti,ab,kw OR 'arterial chemoembolization':ti,ab,kw OR chemoembolisation:ti,ab,kw OR 'transarterial chemoembolisation':ti,ab,kw OR 'transcatheter arterial chemoembolisation':ti,ab,kw OR 'transcatheter chemoembolisation':ti,ab,kw OR 'transcatheter chemoembolization':ti,ab,kw OR 'transcatheter oily chemoembolisation':ti,ab,kw OR 'transcatheter oily chemoembolization':ti,ab,kw OR tace:ti,ab,kw |
| #4 | 'liver cell carcinoma'/exp OR 'hepatocellular carcinomas':ti,ab,kw OR 'hepatocellular carcinoma':ti,ab,kw OR 'liver cell carcinoma':ti,ab,kw OR 'liver cell carcinomas':ti,ab,kw OR 'hepatic cancer':ti,ab,kw OR 'hepatocellular cancer':ti,ab,kw OR 'hepatic malignancy':ti,ab,kw OR 'hepatic tumor':ti,ab,kw OR 'liver cancer':ti,ab,kw OR 'liver carcinoma':ti,ab,kw OR 'liver malignancy':ti,ab,kw OR 'liver tumor':ti,ab,kw OR 'hepatocellular malignancy':ti,ab,kw OR 'cancer of liver':ti,ab,kw OR 'malignancy of liver':ti,ab,kw OR 'carcinoma of liver':ti,ab,kw OR 'hepatic neoplasm':ti,ab,kw OR 'neoplasm of liver':ti,ab,kw OR 'hepatocellular neoplasm':ti,ab,kw OR 'hepatocellular neoplasia':ti,ab,kw OR 'liver neoplasia':ti,ab,kw OR 'hepatic neoplasia':ti,ab,kw OR hepatoma:ti,ab,kw OR hepatomas:ti,ab,kw OR hcc:ti,ab,kw |
| #5 | (#1 AND #2 OR (#1 AND #3) OR (#2 AND #3) OR (#1 AND #2 AND #3)) AND #4 |
| #6 | 'randomized controlled trial'/exp OR 'controlled clinical trial'/exp OR 'controlled study'/exp OR 'case control study'/exp OR 'cohort analysis'/exp OR 'randomized controlled trial' OR 'randomised controlled study' OR 'randomised controlled trial' OR 'randomized controlled study' OR 'controlled clinical trial' OR 'randomized trial' OR 'random*' OR 'control*' OR 'compared' OR 'groups' OR 'cohort' OR 'case control*' OR 'propensity score' OR 'matched' OR 'cohort analyses' |
| #7 | #5 AND #6 |
| Cochrane | |
| #1 | MeSH descriptor: [Hepatectomy] explode all trees |
| #2 | (Hepatectomies):ti,ab,kw OR (hepatic resection):ti,ab,kw OR (liver resection):ti,ab,kw OR (hepatic resection):ti,ab,kw OR (resection):ti,ab,kw |
| #3 | #1 OR #2 |
| #4 | MeSH descriptor: [Radiofrequency Ablation] explode all trees |
| #5 | (radio frequency ablation):ti,ab,kw OR (Radiofrequency):ti,ab,kw OR (intervention ablation):ti,ab,kw OR (Radiofrequency Ablation):ti,ab,kw OR (RFA):ti,ab,kw |
| #6 | #4 OR #5 |
| #7 | MeSH descriptor: [Chemoembolization, Therapeutic] explode all trees |
| #8 | (transcatheter arterial chemoembolization):ti,ab,kw OR (transarterial chemoembolization):ti,ab,kw OR (Embolization):ti,ab,kw OR (chemoembolization):ti,ab,kw OR (TACE):ti,ab,kw |
| #9 | #7 OR #8 |
| #10 | MeSH descriptor: [Carcinoma, Hepatocellular] explode all trees |
| #11 | (hepatocellular carcinomas):ti,ab,kw OR (hepatocellular carcinoma):ti,ab,kw OR (liver cell carcinoma):ti,ab,kw OR (liver cancer):ti,ab,kw OR (HCC):ti,ab,kw |
| #12 | #10 OR #11 |
| #13 | (#3 AND #6 OR (#3 AND #9) OR (#6 AND #9) OR (#3 AND #6 AND #9)) AND #12 |

**Supplementary Table 2.** Details of various studies included in meta-analysis.

| Study | Type of study | Year and country | Arm | treatment | No. of  patients | Age (Mean  or Median) | Sex  (M: F) | Follow-up period  (month) | Follow- up methods | Child-Pugh | BCLC | ECOG | HBV  /HCV  /Others | Mean  Tumor size  （cm） | Tumor  number | Overall survival (%) | Recurrence-free survival (%) |
| --- | --- | --- | --- | --- | --- | --- | --- | --- | --- | --- | --- | --- | --- | --- | --- | --- | --- |
| Li | PSM | China | LR | NA | 58 | 61.0 | 39:19 | 56.0 | US, CT, MRI, biochemical tests | A:56 | 0:58 | NA | 28/34/NA | 1.90 | 1 | 1year:98.2  3year:88.8  5year:77.7 | 1year:93.0  3year:71.7  5year:57.1 |
|  |  | 2021 | RFA | NA | 58 | 61.0 | 39:19 | 56.0 | US, CT, MRI, biochemical tests | A:57 | 0:58 | NA | 23/27/NA | 1.80 | 1 | 1year:91.4  3year:77.2  5year:60.1 | 1year:91.2  3year:62.0  5year:53.2 |
| Lee | PSM | Korea | LR | laparoscopic | 118 | 59.5 | 91:27 | 30.0 | CT or MRI, biochemical tests | A:118 | NA | NA | 90/10/18 | 1.84 | 1 | NA | 1year:87.0  3year:65.5 |
|  |  | 2021 | RFA | Percutaneous  (US) | 118 | 60.5 | 88:30 | 28.0 | CT or MRI, biochemical tests | A:118 | NA | NA | 84/12/22 | 1.87 | 1 | NA | 1year:85.1  3year:62.9 |
| Pan | PSM | China | LR | laparoscopic | 118 | 53.0 | 101:17 | 26.2 | NA | A or B | NA | 0 | NA | 2.50 | ≤3 | 1year:97.3  3year:91.0 | 1year:81.5  3year:56.9 |
|  |  | 2020 | RFA | Percutaneous  (US) | 236 | 56.0 | 206:30 | 24.2 | NA | A or B | NA | 0 | NA | 2.55 | ≤3 | 1year:99.5  3year:79.0 | 1year:62.7  3year:39.9 |
| Chong | PSM | China | LR | laparoscopic or  robotic | 59 | 57.7 | 46:13 | 47.3 | US or CT | A:59  B:0 | 0:23  A:36 | NA | 48/4/- | 2.00 | ≤3 | 1year:94.9  3year:88.2  5year:82.5 | 1year:86.3  3year:68.0  5year:68.0 |
|  |  | 2020 | RFA | laparoscopic or percutaneous | 59 | 59.3 | 46:13 | 47.3 | US or CT | A:58  B:1 | 0:24  A:35 | NA | 48/4/- | 2.30 | ≤3 | 1year:96.6  3year:78.7  5year:53.3 | 1year:59.3  3year:25.3  5year:15.9 |
| Ye | PSM | China | LR | anatomical or non-  anatomical | 154 | ≤60:103  ＞60:51 | 141:13 | NA | CT or MRI, biochemical tests | A:139  B:15 | NA | NA | 135/2/- | 3-4:113  4-5:41 | 1 | 1year:93.0  3year:63.0  5year:40.0 | 1year:76.0  3year:30.0  5year:15.0 |
|  |  | 2019 | RFA | Percutaneous  (US) | 154 | ≤60:103  ＞60:51 | 134:20 | NA | CT or MRI, biochemical tests | A:144  B:10 | NA | NA | 134/5/- | 3-4:111  4-5:43 | 1 | 1year:95.0  3year:58.0  5year:34.0 | 1year:72.0  3year:30.0  5year:10.0 |
| Kim. T. H | PSM | Korea | LR | anatomical or non-  anatomical | 48 | 56.2 | 38:10 | 59.1 | CT or MRI, biochemical tests, PET,  X-ray | A:48 | 0:48 | 0:48 | 36/5/7 | 1.57 | 1 | 1year:97.9  3year:88.8  5year:77.0 | 1year:89.6  3year:57.7  5year:54.7 |
|  |  | 2019 | RFA | Percutaneous  (US) | 48 | 58.7 | 35:13 | 63.3 | CT or MRI, biochemical tests, PET,  X-ray | A:48 | 0:48 | 0:48 | 34/8/6 | 1.53 | 1 | 1year:97.9  3year:91.6  5year:79.2 | 1year:87.3  3year:50.5  5year:29.9 |
| Lee. H. W | RCT | Korea | LR | anatomical | 29 | 55.6 | 23:6 | 60.0 | CT or MRI, biochemical tests | A:29 | NA | NA | NA | 2-4 | 1 | 3year: 96.6  5year: 83.4 | 3year:66.7  5year:44.4 |
|  |  | 2018 | RFA | Percutaneous  (US or CT) | 34 | 56.1 | 24:10 | 60.0 | CT or MRI, biochemical tests | A:34 | NA | NA | NA | 2-4 | 1 | 3year: 97.1  5year: 86.2 | 3year:44.1  5year:31.2 |
| Ng. K.K.C | RCT | China | LR | open approach | 109 | 55.0 | 89:20 | 93.0 | CT  biochemical tests | A:107  B:2 | NA | NA | 99/5/- | 2.90 | ≤3 | 1year: 94.5  3year: 80.6  5year: 66.5 | 1year: 74.1  3year: 50.9  5year: 41.5 |
|  |  | 2017 | RFA | Percutaneous  (US) | 109 | 57.0 | 86:23 | 93.0 | CT  biochemical tests | A:104  B:5 | NA | NA | 95/0/- | 2.60 | ≤3 | 1year: 95.4  3year: 82.3  5year: 66.4 | 1year: 70.6  3year: 46.6  5year: 33.6 |
| Song | PSM | China | LR | laparoscopic | 78 | 48.0 | 70:8 | 31.2 | US, CT, X-ray, biochemical tests | A:78  B:0 | 0:33  A:45 | 0:76  1:1  2:1 | 73/-/- | ＜4 | 1 | 1year:96.2  3year:84.1 | 1year:82.1  3year:60.0 |
|  |  | 2016 | RFA | Percutaneous  (US) | 78 | 48.0 | 70:8 | 31.2 | US, CT, X-ray, biochemical tests | A:76  B:2 | 0:40  A:38 | 0:75  1:3  2:0 | 77/-/- | ＜4 | 1 | 1year:96.2  3year:78.8 | 1year:65.4  3year:37.6 |
| Liu | PSM | China | LR | anatomical | 79 | 61.0 | 55:24 | 43.0 | NA | A:79 | NA | 0:69  1:10 | 46/31/7 | ≤2 | 1 | 1year:97.0  3year:97.0  5year:80.0 | 1year:92.0  3year:65.0  5year:48.0 |
|  |  | 2016 | RFA | Percutaneous  (US) | 79 | 63.0 | 52:27 | 44.0 | NA | A:79 | NA | 0:68  1:11 | 36/30/9 | ≤2 | 1 | 1year:97.0  3year:83.0  5year:66.0 | 1year:68.0  3year:36.0  5year:18.0 |
| Kang. T. W | PSM | Korea | LR | non-  anatomical | 99 | 54.0 | 77:22 | 59.7 | CT, X-ray, biochemical tests | A:95  B:4 | 0:46  A:53 | 0 | 83/8/4 | 2.00 | 1 | 3year:92.7  5year:90.2 | 3year:67.1  5year:54.4 |
|  |  | 2015 | RFA | Percutaneous  (US) | 99 | 55.0 | 77:22 | 50.9 | CT, X-ray, biochemical tests | A:95  B:4 | 0:50  A:49 | 0 | 83/8/4 | 1.90 | 1 | 3year:95.7  5year:87.2 | 3year:53.3  5year:48.9 |
| Jiang | PSM | China | LR | open approach | 140 | 53.0 | 123:17 | 29.8 | US, CT, MRI, biochemical tests | A:139  B:1 | A:140 | 0 | 129/NA/NA | 2.40 | ≤3 | 1year:95.0  3year:72.9  5year:38.9 | 1year:84.3  3year:52.4  5year:18.9 |
|  |  | 2015 | RFA | Percutaneous  laparoscopic  open methods | 140 | 55.0 | 118:22 | 29.8 | US, CT, MRI, biochemical tests | A:135  B:5 | A:140 | 0 | 121/NA/NA | 2.30 | ≤3 | 1year:90.0  3year:74.8  5year:38.9 | 1year:85.0  3year:35.8  5year:10.1 |
| Fang | RCT | China | LR | anatomical or non-  anatomical | 60 | 53.5 | 46:14 | 40.0 | X-ray, CT, MRI, biochemical tests | A:43  B:17 | NA | NA | 52/NA/NA | 2.28 | ≤3 | 1year:93.7  3year:77.5 | 1year:90.4  3year:41.3 |
|  |  | 2014 | RFA | Percutaneous  (US or CT) | 60 | 51.4 | 42:18 | 40.0 | X-ray, CT, MRI, biochemical tests | A:32  B:23  C:5 | NA | NA | 55/NA/NA | 2.21 | ≤3 | 1year:97.5  3year:82.5 | 1year:91.6  3year:55.4 |
| Pompili | PSM | Italy | LR | anatomical or non-  anatomical | 116 | 67.0 | 87:29 | 41.0 | US, CT, MRI, biochemical tests | A:116 | NA | NA | 11/78/26 | 2.30 | 1 | 1year:95.5  3year:76.0 | NA |
|  |  | 2013 | RFA | NA | 116 | 69.0 | 92:24 | 38.0 | US, CT, MRI, biochemical tests | A:116 | NA | NA | 17/78/21 | 2.30 | 1 | 1year:97.3  3year:78.1 | NA |
| Feng | RCT | China | LR | anatomical | 84 | 47.0 | 75:9 | 36.0 | X-ray, CT, MRI, biochemical tests | A:43  B:41 | NA | NA | NA | 2.60 | ≤2 | 1year:96.0  3year:74.8 | 1year:90.6  3year:61.1 |
|  |  | 2012 | RFA | Percutaneous  (US) | 84 | 51.0 | 79:5 | 36.0 | X-ray, CT, MRI, biochemical tests | A:39  B:45 | NA | NA | NA | 2.40 | ≤2 | 1year:93.1  3year:67.2 | 1year:86.2  3year:49.6 |
| Huang | RCT | China | LR | anatomical | 115 | 55.9 | 85:30 | 60.0 | US, CT, MRI, biochemical tests | A:106  B:9 | NA | NA | 104/6/5 | ≤5 | ≤3 | 1year:98.3  3year:92.2  5year:75.7 | 1year:85.2  3year:60.9  5year:51.3 |
|  |  | 2010 | RFA | Percutaneous  (US) | 115 | 56.6 | 79:36 | 60.0 | US, CT, MRI, biochemical tests | A:110  B:5 | NA | NA | 101/4/10 | ≤5 | ≤3 | 1year:87.0  3year:69.6  5year:54.8 | 1year:81.7  3year:46.1  5year:28.7 |
| Chen | RCT | China | LR | anatomical | 90 | 49.4 | 75:15 | 29.2 | X-ray, CT, MRI, biochemical tests | A:90 | NA | NA | NA | ≤5 | 1 | 1year:93.3  3year:73.4 | 1year: 86.6  3year: 69.0 |
|  |  | 2006 | RFA | Percutaneous | 71 | 51.9 | 56:15 | 27.9 | X-ray, CT, MRI,  biochemical tests | A:71 | NA | NA | NA | ≤5 | 1 | 1year:95.8  3year:71.4 | 1year: 85.9  3year: 64.1 |
| Lee. H. J | PSM | Korea | LR | anatomical | 26 | 59.6 | 22:4 | 48.4 | CT or MRI, biochemical tests | A:26  B:0 | A:26 | 0 | 13/7/10 | 3.58 | 1 | 1year:96.2  3year:91.3  5year:62.1 | 1year:88.5  3year:48.2  5year:26.8 |
|  |  | 2019 | RFA+  TACE | Percutaneous  (US)  C-TACE | 26 | 62.4 | 21:5 | 41.9 | CT or MRI, biochemical tests | A:25  B:1 | A:26 | 0 | 14/6/6 | 3.60 | 1 | 1year:100.0  3year:87.5  5year:56.5 | 1year:100.0  3year:71.2  5year:33.4 |
| Lee. H. J | PSM | Korea | LR | laparoscopic or  Open | 49 | 60.8 | 37:12 | 41.0 | CT or MRI, biochemical tests | A:49 | A:49 | NA | 33/7/8 | 2.47 | 1 | 1year:93.9  3year:86.7  5year:74.6 | 1year:83.7  3year:63.4  5year:45.4 |
|  |  | 2017 | RFA+  TACE | NA(US)  C-TACE | 49 | 61.7 | 37:12 | 34.3 | CT or MRI, biochemical tests | A:49 | A:49 | NA | 36/3/7 | 2.55 | 1 | 1year:95.9  3year:87.4  5year:87.4 | 1year:91.7  3year:63.1  5year:55.2 |
| Bholee | PSM | China | LR | anatomical or non-  anatomical | 148 | 52.2 | 136:12 | 50.2 | CT or MRI, biochemical tests | A:144  B:4 | NA | NA | 135/2/NA | 3.00 | ≤3 | 1year:91.2  3year:64.4  5year:47.7 | 1year:68.9  3year:49.2  5year:40.9 |
|  |  | 2017 | RFA+  TACE | Percutaneous  (US)  C-TACE | 74 | 54.9 | 68:6 | 56.9 | CT or MRI, biochemical tests | A:70  B:4 | NA | NA | 70/4/NA | 2.90 | ≤3 | 1year:94.6  3year:75.1  5year:55.3 | 1year:87.8  3year:48.3  5year:33.5 |
| H. Liu | RCT | China | LR | anatomical | 100 | 49.0 | 94:6 | 56.0 | X-ray, CT, MRI,  biochemical tests | A:98  B:2 | NA | NA | 90/NA/NA | 3.00 | ≤3 | 1year: 97.0  3year: 83.7  5year: 61.9 | 1year: 94.0  3year:68.2  5year:48.4 |
|  |  | 2016 | RFA+  TACE | Percutaneous  (US)  C-TACE | 100 | 52.0 | 86:14 | 56.0 | X-ray, CT, MRI,  biochemical tests | A:96  B:4 | NA | NA | 87/NA/NA | 2.80 | ≤3 | 1year: 96.0  3year: 67.2  5year: 45.7 | 1year:83.0  3year:44.9  5year:35.5 |
| Takuma | PSM | Japan | LR | anatomical | 75 | 70.0 | 48:27 | 45.0 | US, CT, MRI, biochemical tests | A:69  B:6 | NA | NA | 5/60/NA | 2.30 | ≤3 | 1year:95.0  3year:87.0  5year:75.0 | 1year:79.0  3year:53.0  5year:32.0 |
|  |  | 2013 | RFA+  TACE | Percutaneous  (US)  C-TACE | 75 | 70.0 | 56:19 | 45.0 | US, CT, MRI, biochemical tests | A:71  B:4 | N A | NA | 4/62/NA | 2.20 | ≤3 | 1year:99.0  3year:88.0  5year:70.0 | 1year:85.0  3year:35.0  5year:17.0 |
| Zhang | RCT | China | RFA | Percutaneous  (US) | 95 | 55.3 | 71:24 | 50.0 | X-ray, CT, US, biochemical tests | A:90  B:5 | NA | 0 | 83/6/NA | 3.39 | ≤3 | 1year:85.4  3year:57.9  5year:43.2 | 1year:64.2  3year:37.9  5year:27.4 |
|  |  | 2021 | RFA+  TACE | Percutaneous  (US)  C-TACE | 94 | 53.3 | 75:19 | 56.0 | X-ray, CT, US, biochemical tests | A:90  B:4 | NA | 0 | 85/6/NA | 3.47 | ≤3 | 1year:94.9  3year:69.1  5year:52.0 | 1year:78.7  3year:54.3  5year:41.4 |
| Morimoto | RCT | Japan | RFA | Percutaneous  (US) | 18 | 73.0 | 12:6 | 32.0 | CT, US, biochemical tests | A:16  B:2 | NA | 0:12  1:6 | 0/16/2 | 3.70 | 1 | 1year:89.0  3year:80.0  5year:69.0 | NA |
|  |  | 2010 | RFA+  TACE | Percutaneous  (US)  C-TACE | 19 | 70.0 | 15:4 | 30.0 | CT, US, biochemical tests | A:18  B:1 | NA | 0:12  1:7 | 0/17/2 | 3.60 | 1 | 1year:100.0  3year:93.0  5year:68.0 | NA |
| Shibata | RCT | Japan | RFA | Percutaneous  (US or CT) | 43 | 69.8 | 33:10 | 30.4 | CT,  biochemical tests | A:33  B:10 | NA | NA | 9/30/NA | 1.60 | ≤3 | 1year: 100.0  3year: 84.5 | 1year: 74.3  3year: 29.7 |
|  |  | 2009 | RFA+  TACE | Percutaneous  (US or CT)  C-TACE | 46 | 67.2 | 31:15 | 30.4 | CT,  biochemical tests | A:32  B:14 | NA | NA | 12/32/NA | 1.70 | ≤3 | 1year: 100.0  3year: 84.8 | 1year: 71.3  3year: 48.8 |

**Supplementary Table 3.** Rank probability table for all studies.

|  | **Treatment** | **Rank 1** | **Rank 2** | **Rank 3** | **SUCRA** |
| --- | --- | --- | --- | --- | --- |
| **OS at 1 year** | **LR** | 0.04 | 0.69 | 0.28 | 0.38 |
|  | **RFA** | 0.02 | 0.28 | 0.70 | 0.16 |
|  | **RFA+TACE** | 0.94 | 0.04 | 0.02 | 0.96 |
| **OS at 3 years** | **LR** | 0.57 | 0.42 | 0.01 | 0.78 |
|  | **RFA** | 0.01 | 0.13 | 0.87 | 0.07 |
|  | **RFA+TACE** | 0.42 | 0.46 | 0.12 | 0.65 |
| **OS at 5 years** | **LR** | 0.68 | 0.32 | 0.01 | 0.84 |
|  | **RFA** | 0.01 | 0.09 | 0.90 | 0.05 |
|  | **RFA+TACE** | 0.32 | 0.59 | 0.09 | 0.61 |
| **RFS at 1 year** | **LR** | 0.22 | 0.77 | 0.01 | 0.61 |
|  | **RFA** | 0.00 | 0.03 | 0.97 | 0.01 |
|  | **RFA+TACE** | 0.77 | 0.21 | 0.02 | 0.88 |
| **RFS at 3 years** | **LR** | 0.79 | 0.21 | 0.00 | 0.89 |
|  | **RFA** | 0.00 | 0.05 | 0.95 | 0.02 |
|  | **RFA+TACE** | 0.21 | 0.74 | 0.05 | 0.58 |
| **RFS at 5 years** | **LR** | 0.83 | 0.17 | 0.00 | 0.91 |
|  | **RFA** | 0.00 | 0.03 | 0.97 | 0.02 |
|  | **RFA+TACE** | 0.17 | 0.79 | 0.03 | 0.57 |

**Supplementary Table 4.** Model diagnostics of the developed consistency model for the outcomes of the study.

| **Outcome** | **Residual deviance (Dbar)** | **Leverage (PD)** | **Deviance Information Criterion (DIC)** | **Number of data points** | **I^2^** |
| --- | --- | --- | --- | --- | --- |
| **OS at 1 year** | 20.4 | 6.33 | 26.71 | 22 | 0% |
| **OS at 3 years** | 24.7 | 12.9 | 37.6 | 24 | 7% |
| **OS at 5 years** | 17.6 | 9.5 | 27.1 | 17 | 9% |
| **RFS at 1 year** | 23.0 | 12.3 | 35.3 | 21 | 13% |
| **RFS at 3 years** | 24.8 | 15.1 | 39.8 | 23 | 11% |
| **RFS at 5 years** | 18.1 | 11.5 | 29.6 | 16 | 17% |

**Supplementary Table 5.** League Table of subgroup analysis (Single tumor studies).

|  | **Treatment** | **LR** | **RFA** | **RFA+TACE** |
| --- | --- | --- | --- | --- |
| **OS at 1 year** | **LR vs** | NA | 1.12(0.52,2.21) | 2.25(0.38,13.77) |
|  | **RFA vs** | 0.90 (0.45, 1.92) | NA | 2.02(0.32,13.79) |
|  | **RFA+TACE vs** | 0.44 (0.07, 2.60) | 0.50 (0.07, 3.11) | NA |
| **OS at 3 years** | **LR vs** | NA | 0.82 (0.56, 1.14) | 1.12 (0.44, 2.99) |
|  | **RFA vs** | 1.22 (0.88, 1.79) | NA | 1.36 (0.52, 3.85) |
|  | **RFA+TACE vs** | 0.90 (0.33, 2.26) | 0.74 (0.26, 1.92) | NA |
| **OS at 5 years** | **LR vs** | NA | 0.78 (0.55, 1.12) | 1.14 (0.58, 2.20) |
|  | **RFA vs** | 1.29 (0.89, 1.83) | NA | 1.46 (0.72, 2.97) |
|  | **RFA+TACE vs** | 0.88 (0.45, 1.72) | 0.68 (0.34, 1.39) | NA |
| **RFS at 1 year** | **LR vs** | NA | 0.65 (0.39, 1.06) | 2.52(0.61,11.57) |
|  | **RFA vs** | 1.53 (0.94, 2.59) | NA | 3.87(0.86,19.59) |
|  | **RFA+TACE vs** | 0.40 (0.09, 1.65) | 0.26 (0.05, 1.16) | NA |
| **RFS at 3 years** | **LR vs** | NA | 0.70 (0.53, 0.90) | 1.34 (0.68, 2.72) |
|  | **RFA vs** | 1.42 (1.11, 1.88) | NA | 1.91 (0.93, 4.12) |
|  | **RFA+TACE vs** | 0.75 (0.37, 1.48) | 0.52 (0.24, 1.08) | NA |
| **RFS at 5 years** | **LR vs** | NA | 0.68 (0.49, 0.93) | 1.27 (0.68, 2.34) |
|  | **RFA vs** | 1.46 (1.07, 2.04) | NA | 1.85 (0.92, 3.72) |
|  | **RFA+TACE vs** | 0.79 (0.43, 1.48) | 0.54 (0.27, 1.08) | NA |

**Supplementary Table 6.** Rank probability table for subgroup analysis (Single tumor studies).

|  | **Treatment** | **Rank1** | **Rank2** | **Rank3** | **SUCRA** |
| --- | --- | --- | --- | --- | --- |
| **OS at 1 year** | **LR** | 0.07 | 0.40 | 0.52 | 0.27 |
|  | **RFA** | 0.17 | 0.51 | 0.32 | 0.42 |
|  | **RFA+TACE** | 0.76 | 0.09 | 0.15 | 0.80 |
| **OS at 3 years** | **LR** | 0.37 | 0.57 | 0.06 | 0.65 |
|  | **RFA** | 0.04 | 0.28 | 0.68 | 0.18 |
|  | **RFA+TACE** | 0.58 | 0.16 | 0.26 | 0.66 |
| **OS at 5 years** | **LR** | 0.34 | 0.61 | 0.05 | 0.64 |
|  | **RFA** | 0.03 | 0.16 | 0.81 | 0.11 |
|  | **RFA+TACE** | 0.64 | 0.22 | 0.14 | 0.75 |
| **RFS at 1 year** | **LR** | 0.09 | 0.87 | 0.03 | 0.53 |
|  | **RFA** | 0.01 | 0.06 | 0.93 | 0.04 |
|  | **RFA+TACE** | 0.90 | 0.07 | 0.04 | 0.93 |
| **RFS at 3 years** | **LR** | 0.19 | 0.80 | 0.00 | 0.59 |
|  | **RFA** | 0.00 | 0.04 | 0.96 | 0.02 |
|  | **RFA+TACE** | 0.81 | 0.16 | 0.04 | 0.88 |
| **RFS at 5 years** | **LR** | 0.21 | 0.78 | 0.01 | 0.60 |
|  | **RFA** | 0.00 | 0.04 | 0.96 | 0.02 |
|  | **RFA+TACE** | 0.79 | 0.17 | 0.04 | 0.88 |

**Supplementary Table 7.** League Table of subgroup analysis (Tumor diameter ≤ 3cm).

|  | **Treatment** | **LR** | **RFA** | **RFA+TACE** |
| --- | --- | --- | --- | --- |
| **OS at 1 year** | **LR vs** | NA | 0.97 (0.56, 1.77) | 1.46 (0.61, 3.57) |
|  | **RFA vs** | 1.03 (0.56,1.80) | NA | 1.52 (0.53, 4.22) |
|  | **RFA+TACE vs** | 0.68 (0.28,1.64) | 0.66 (0.24,1.90) | NA |
| **OS at 3 years** | **LR vs** | NA | 0.81 (0.56,1.14) | 0.91 (0.53, 1.57) |
|  | **RFA vs** | 1.23 (0.88,1.79) | NA | 1.13 (0.61, 2.13) |
|  | **RFA+TACE vs** | 1.09 (0.64,1.89) | 0.89 (0.47,1.63) | NA |
| **OS at 5 years** | **LR vs** | NA | 0.71 (0.46,1.07) | 0.98 (0.58, 1.76) |
|  | **RFA vs** | 1.40 (0.94,2.19) | NA | 1.37 (0.72, 2.92) |
|  | **RFA+TACE vs** | 1.02 (0.57,1.72) | 0.73 (0.34,1.39) | NA |
| **RFS at 1 year** | **LR vs** | NA | 0.66 (0.42,1.06) | 1.12 (0.55, 2.22) |
|  | **RFA vs** | 1.51 (0.95,2.39) | NA | 1.68 (0.77, 3.62) |
|  | **RFA+TACE vs** | 0.89 (0.45,1.81) | 0.59 (0.28,1.30) | NA |
| **RFS at 3 years** | **LR vs** | NA | 0.66 (0.51,0.85) | 0.77 (0.52, 1.16) |
|  | **RFA vs** | 1.51 (1.18,1.95) | NA | 1.17 (0.76, 1.84) |
|  | **RFA+TACE vs** | 1.29 (0.86,1.91) | 0.85 (0.54,1.32) | NA |
| **RFS at 5 years** | **LR vs** | NA | 0.59 (0.39,0.86) | 0.81 (0.49, 1.38) |
|  | **RFA vs** | 1.70 (1.17,2.55) | NA | 1.39 (0.74, 2.71) |
|  | **RFA+TACE vs** | 1.23 (0.72,2.03) | 0.72 (0.37,1.35) | NA |

**Supplementary Table 8.** Rank probability table for subgroup analysis (Tumor diameter ≤ 3cm).

|  | **Treatment** | **Rank1** | **Rank2** | **Rank3** | **SUCRA** |
| --- | --- | --- | --- | --- | --- |
| **OS at 1 year** | **LR** | 0.10 | 0.52 | 0.37 | 0.36 |
|  | **RFA** | 0.16 | 0.35 | 0.49 | 0.33 |
|  | **RFA+TACE** | 0.74 | 0.13 | 0.13 | 0.80 |
| **OS at 3 years** | **LR** | 0.58 | 0.38 | 0.04 | 0.77 |
|  | **RFA** | 0.07 | 0.30 | 0.63 | 0.22 |
|  | **RFA+TACE** | 0.35 | 0.32 | 0.33 | 0.51 |
| **OS at 5 years** | **LR** | 0.52 | 0.46 | 0.02 | 0.75 |
|  | **RFA** | 0.03 | 0.14 | 0.83 | 0.10 |
|  | **RFA+TACE** | 0.46 | 0.40 | 0.15 | 0.65 |
| **RFS at 1 year** | **LR** | 0.36 | 0.62 | 0.03 | 0.66 |
|  | **RFA** | 0.02 | 0.09 | 0.89 | 0.06 |
|  | **RFA+TACE** | 0.63 | 0.29 | 0.08 | 0.77 |
| **RFS at 3 years** | **LR** | 0.90 | 0.10 | 0.00 | 0.95 |
|  | **RFA** | 0.00 | 0.22 | 0.78 | 0.11 |
|  | **RFA+TACE** | 0.09 | 0.68 | 0.22 | 0.44 |
| **RFS at 5 years** | **LR** | 0.80 | 0.19 | 0.00 | 0.90 |
|  | **RFA** | 0.00 | 0.13 | 0.87 | 0.07 |
|  | **RFA+TACE** | 0.19 | 0.68 | 0.13 | 0.53 |

| **Supplementary Table 9.** The shared beta coefficients and 95% confidence intervals for the three regression parameters. | | | | | |
| --- | --- | --- | --- | --- | --- |
| **Model** | **Covariate** | **Outcome** | **Input standardisation** | **Centering value** | **Beta(95%CI)** |
| **Regression model** | **Study type** | **OS at 1 year** | (Type - 0.41) / 1 | 0.41 | -0.32(-1.20-0.56) |
|  |  | **OS at 3 years** | (Type - 0.42) / 1 | 0.42 | -0.02(-0.56-0.54) |
|  |  | **OS at 5 years** | (Type - 0.35) / 1 | 0.35 | 0.06(-0.46-0.63) |
|  |  | **RFS at 1 year** | (Type - 0.38) / 1 | 0.38 | 0.43(-0.15-1.02) |
|  |  | **RFS at 3 years** | (Type - 0.39) / 1 | 0.39 | 0.15(-0.22-0.54) |
|  |  | **RFS at 5 years** | (Type - 0.31) / 1 | 0.31 | 0.07(-0.43-0.60) |
| **Regression model** | **year** | **OS at 1 year** | (Year-2015.45) / 8.34 | 2015.45 | 0.18(-0.66-1.04) |
|  |  | **OS at 3 years** | (Year-2015.54)/ 8.04 | 2015.54 | -0.07(-0.57-0.43) |
|  |  | **OS at 5 years** | (Year-2016.65) / 6.63 | 2016.65 | 0.01(-0.49-0.48) |
|  |  | **RFS at 1 year** | (Year-2016.10) / 8.39 | 2016.10 | -0.32(-0.85-0.21) |
|  |  | **RFS at 3 years** | (Year-2016.13) / 8.05 | 2016.13 | -0.06(-0.41-0.29) |
|  |  | **RFS at 5 years** | (Year-2017.06) / 5.86 | 2017.06 | -0.03(-0.50-0.42) |
| **Regression model** | **Sample size** | **OS at 1 year** | (Sample size- 84.77) / 75.40 | 84.77 | 0.03(-1.00-1.07) |
|  |  | **OS at 3 years** | (Sample size- 83.04) / 75.86 | 83.04 | 0.09(-0.54-0.71) |
|  |  | **OS at 5 years** | (Sample size- 82.41) / 84.85 | 82.41 | 0.20(-0.38-0.71) |
|  |  | **RFS at 1 year** | (Sample size- 88.00) / 71.32 | 88.00 | 0.17(-0.53-0.83) |
|  |  | **RFS at 3 years** | (Sample size- 85.91) / 72.53 | 85.91 | 0.23(-0.17-0.62) |
|  |  | **RFS at 5 years** | (Sample size- 86.38) / 80.87 | 86.38 | 0.34(-0.15-0.80) |

**Supplementary Figure 1.** Quality assessment for all studies.

A: Risk of bias of randomized controlled trials.

B: Risk of bias of cohort studies.

**
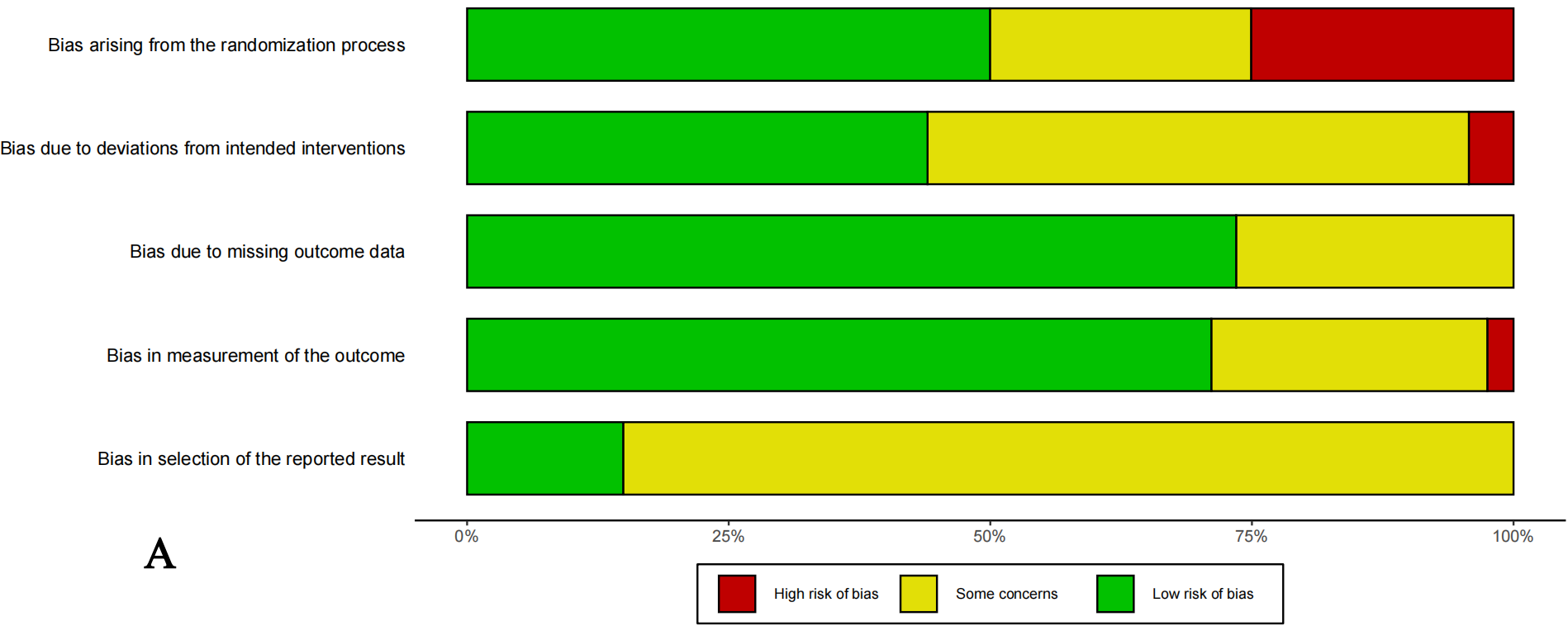
**

**
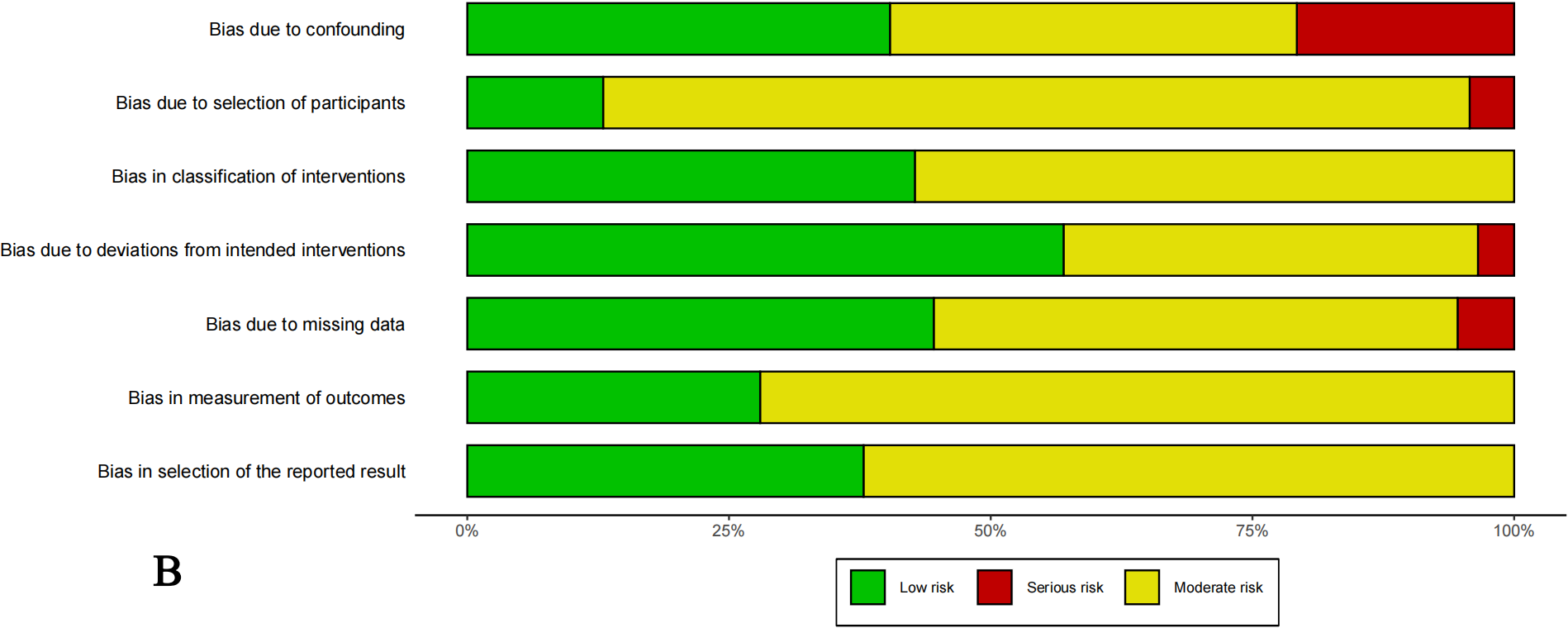
**

**Supplementary Figure 2.** Pairwise meta-analysis: LR vs RFA.

A: 1, 3, and 5-year Overall survival.

B: 1, 3, and 5-year Recurrence-free survival.

**
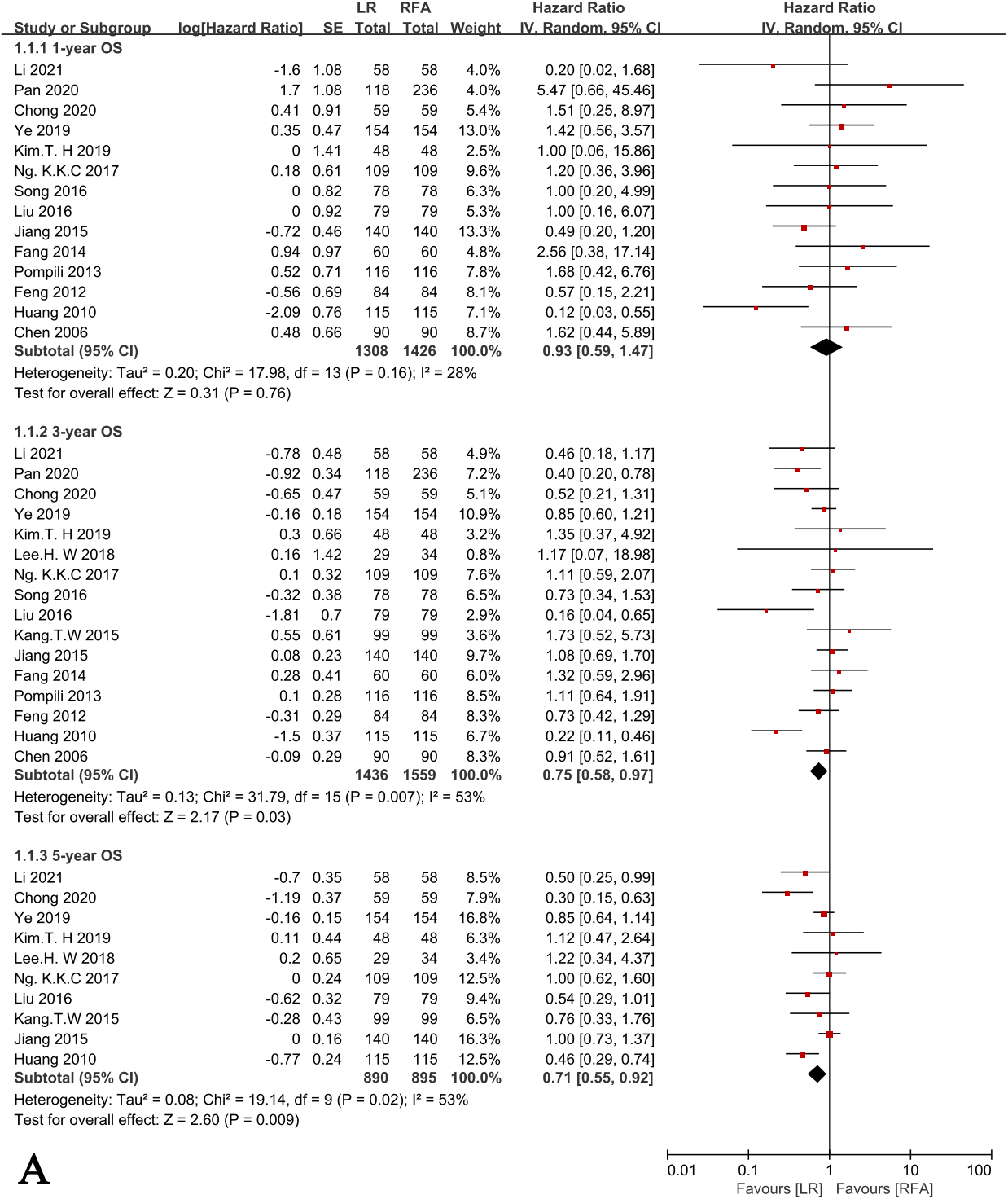
**


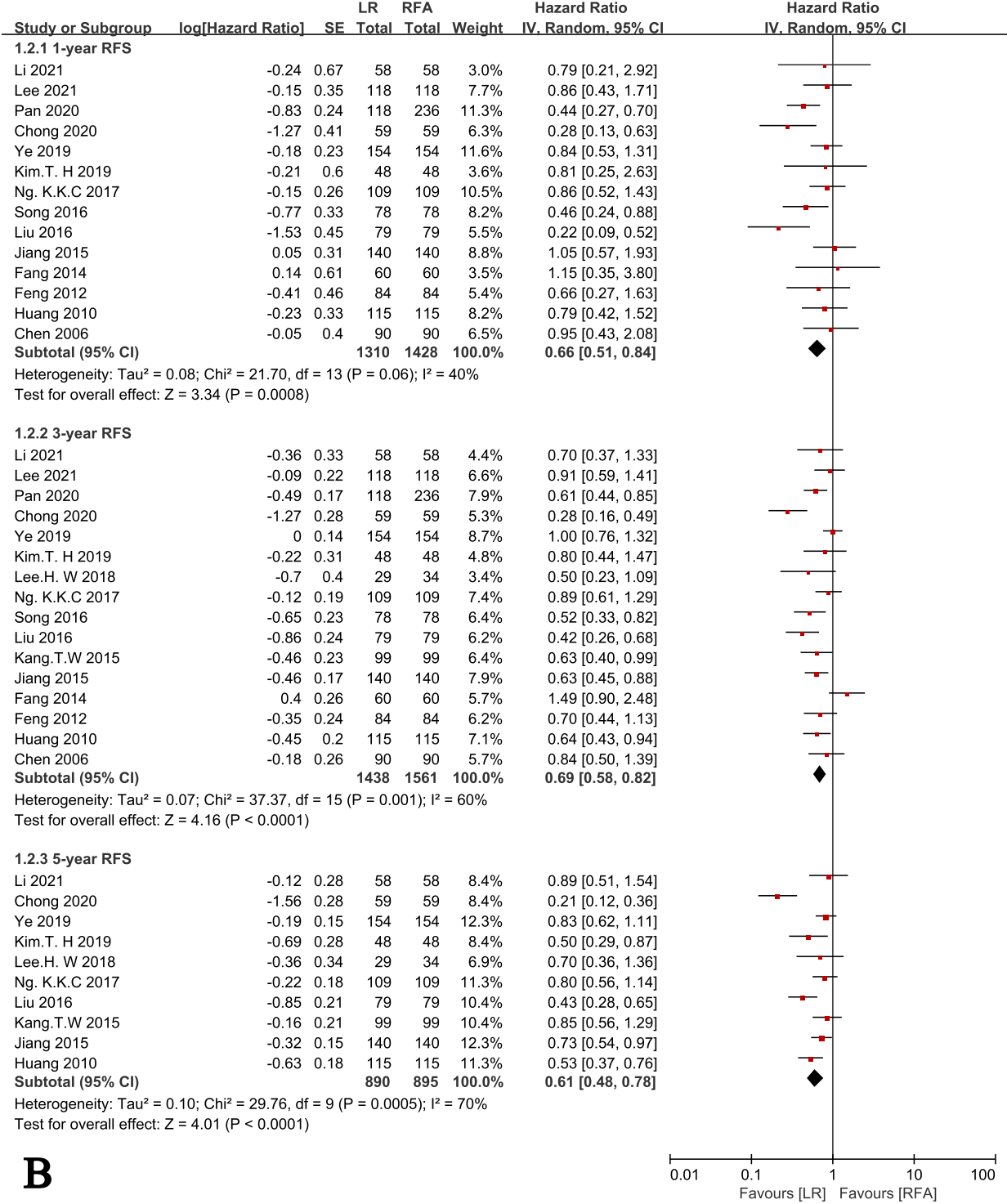


**Supplementary Figure 3.** Pairwise meta-analysis: LR vs RFA+TACE.

A: 1, 3, and 5-year Overall survival.

B: 1, 3, and 5-year Recurrence-free survival.


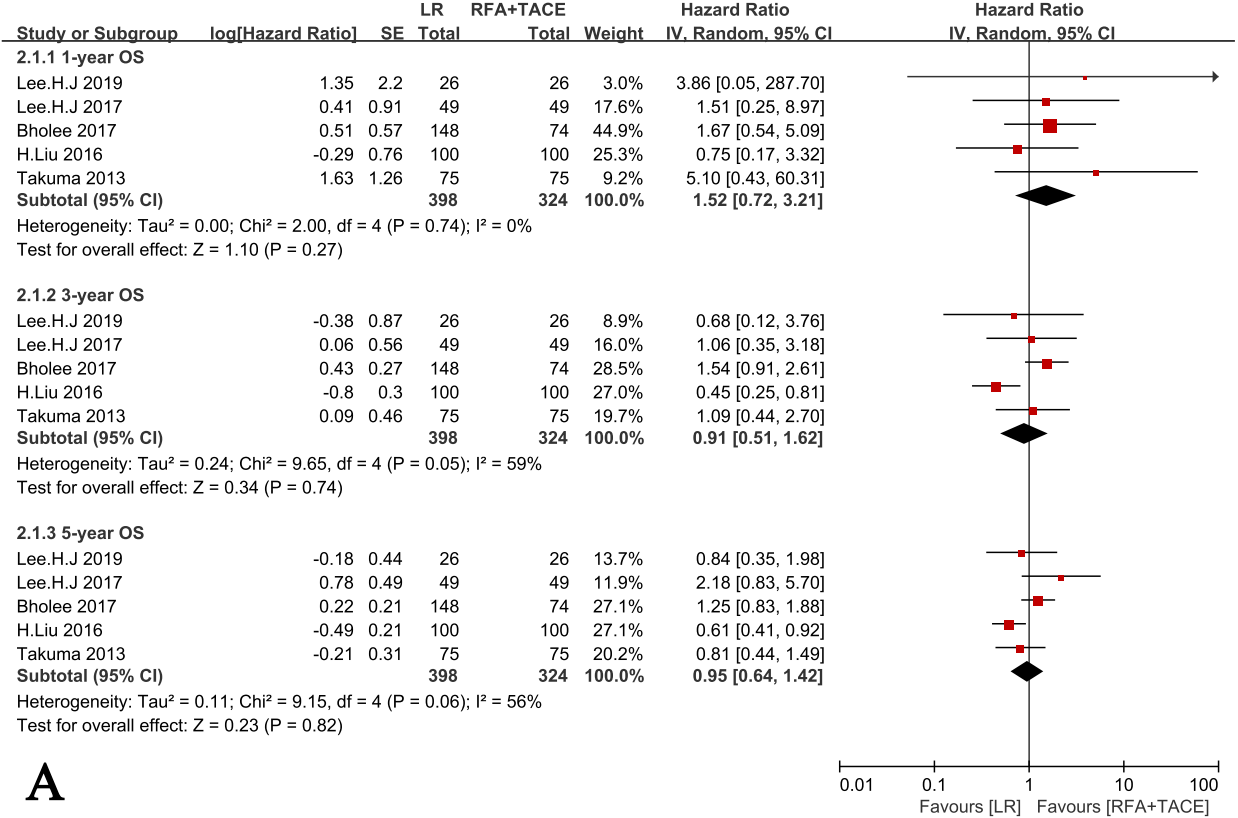


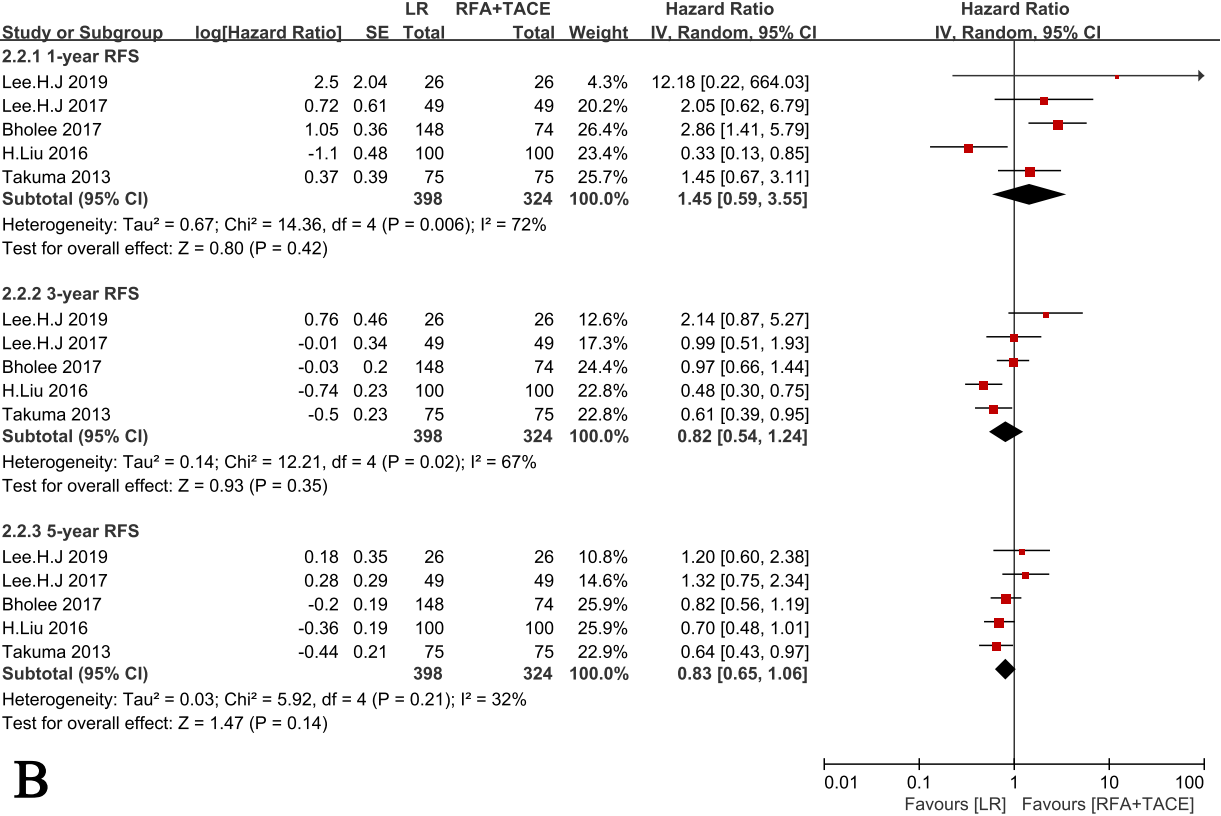


**Supplementary Figure 4.** Pairwise meta-analysis: RFA+TACE vs RFA.

A: 1, 3, and 5-year Overall survival.

B: 1, 3, and 5-year Recurrence-free survival.


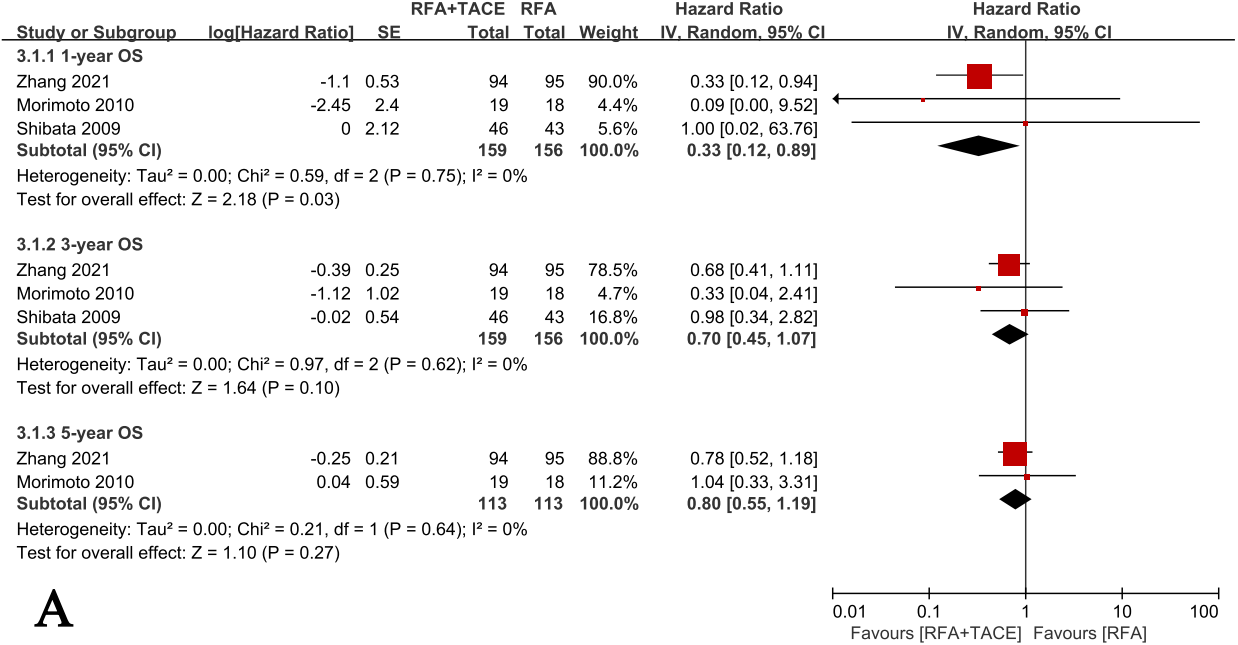


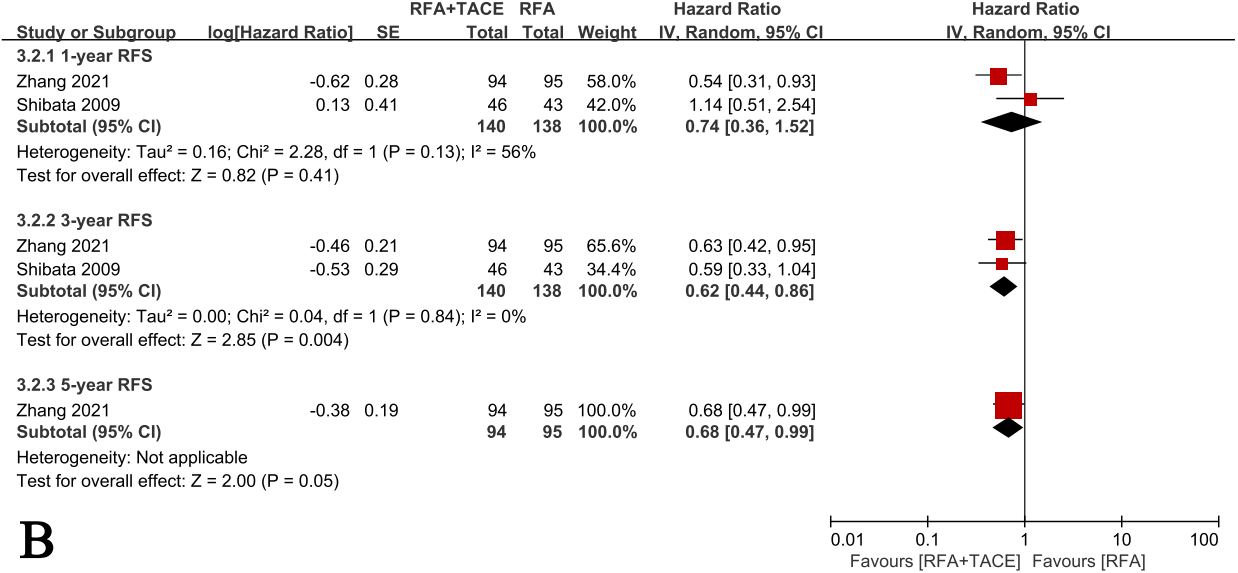


**Supplementary Figure 5**: Graph of network nodes included in the study, the circles represent interventions, and the line thickness between the two treatment nodes indicates the number of comparative studies.


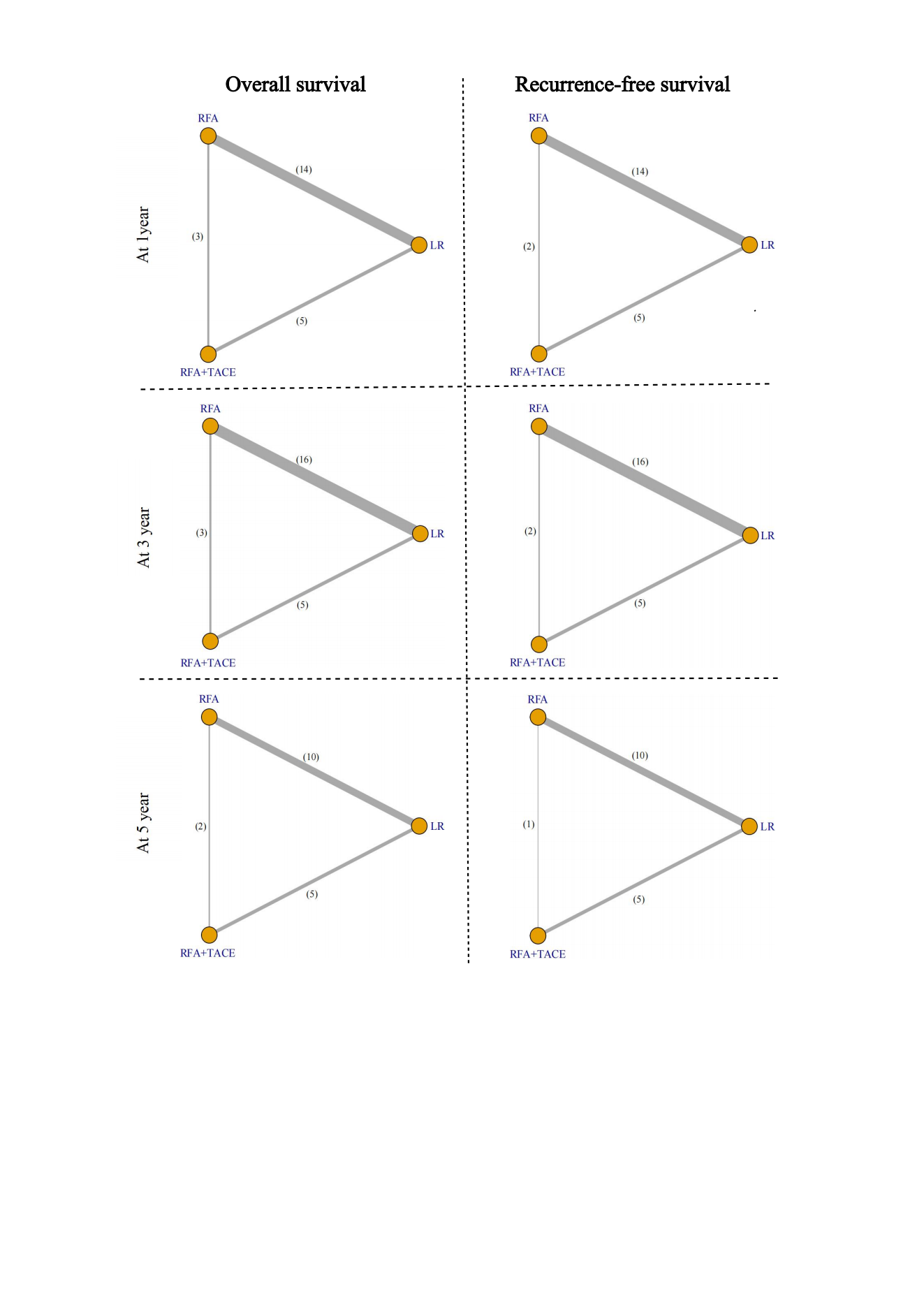


**Supplementary Figure 6.** SUCRA plot for all results.


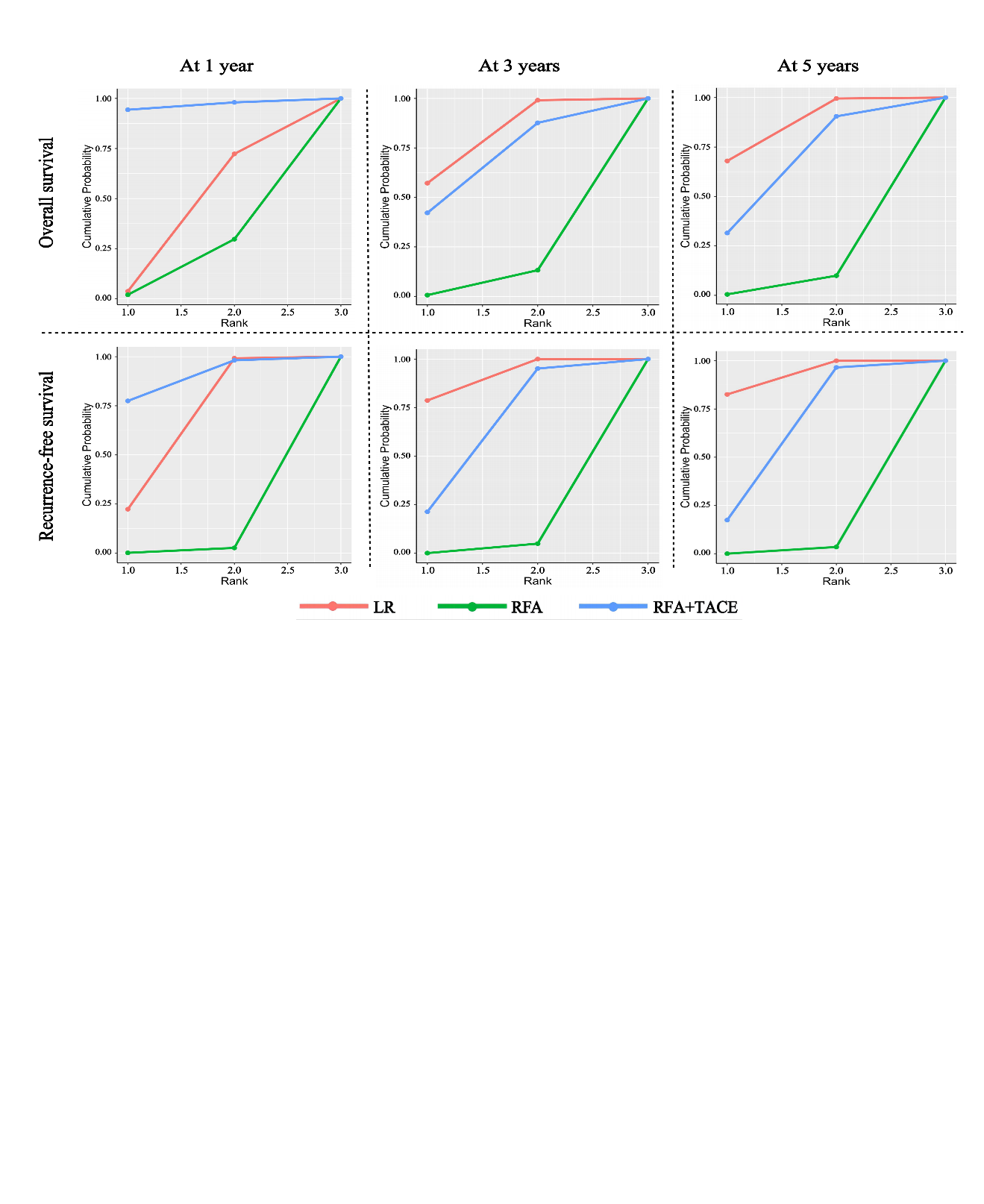


**Supplementary Figure 7. T**o assess publication bias, funnel plots were made for Pairwise meta-analyses involving more than 10 studies.

A: Funnel plots (LR vs RFA): 1, 3, and 5-year Overall survival.

B: Funnel plots (LR vs RFA): 1, 3, and 5-year Recurrence-free survival.


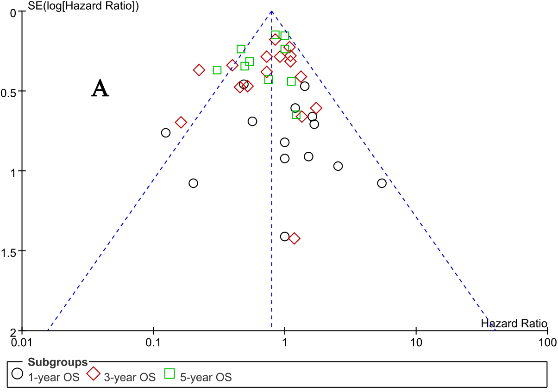

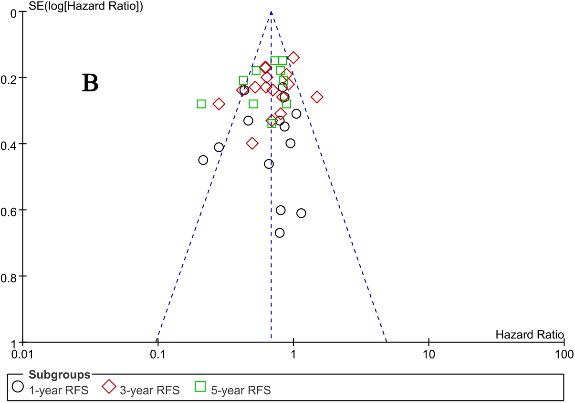


**Supplementary Figure 8.** The inconsistencies of all closed-loop structures are detected by the Node-Splitting method. (All studies)


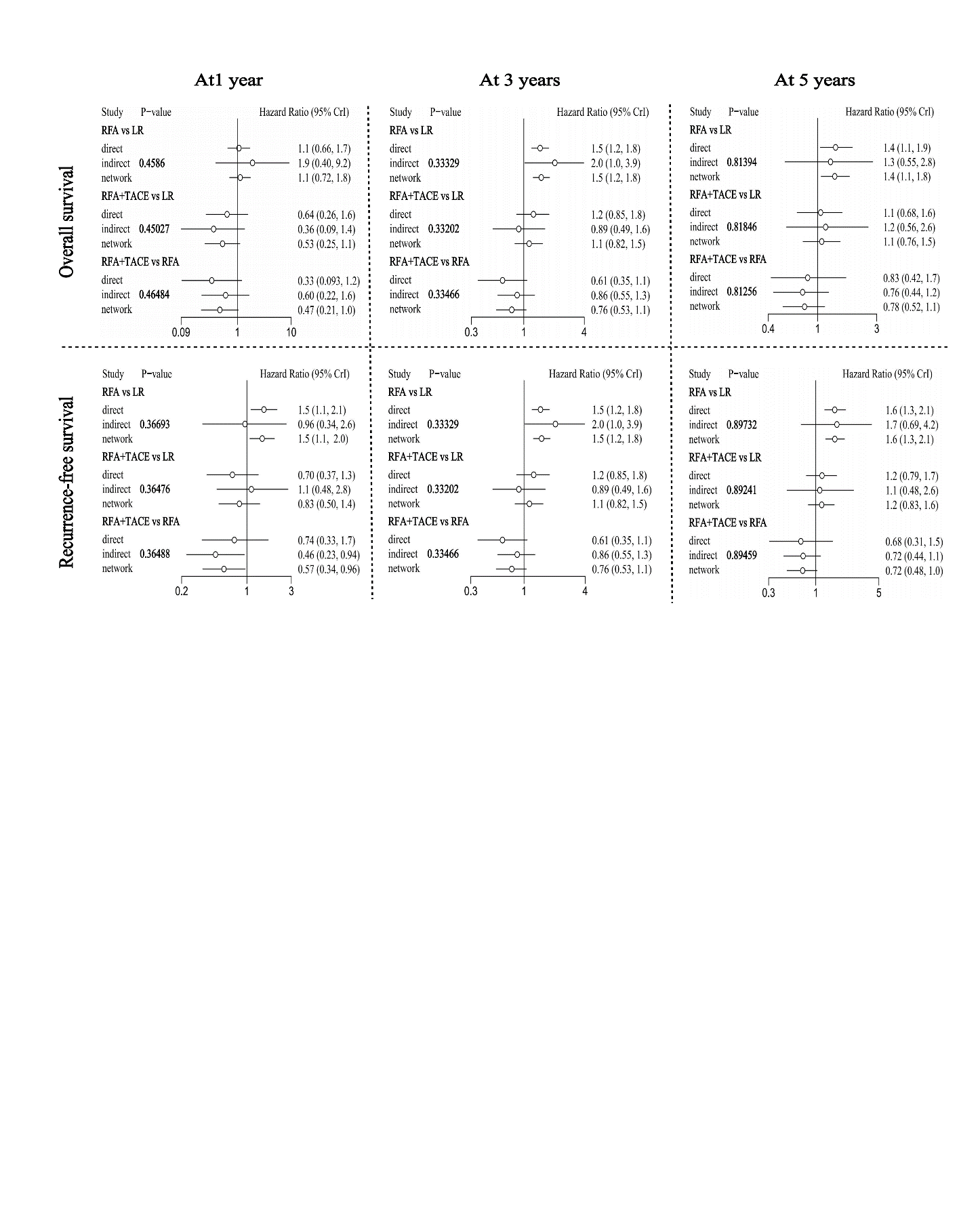


**Supplement Figure 9.** Hazard ratio (for OS and RFS) along with 95% credible interval (CI) for LR and RFA+TACE compared with RFA for the subgroup analysis (Single tumor studies).


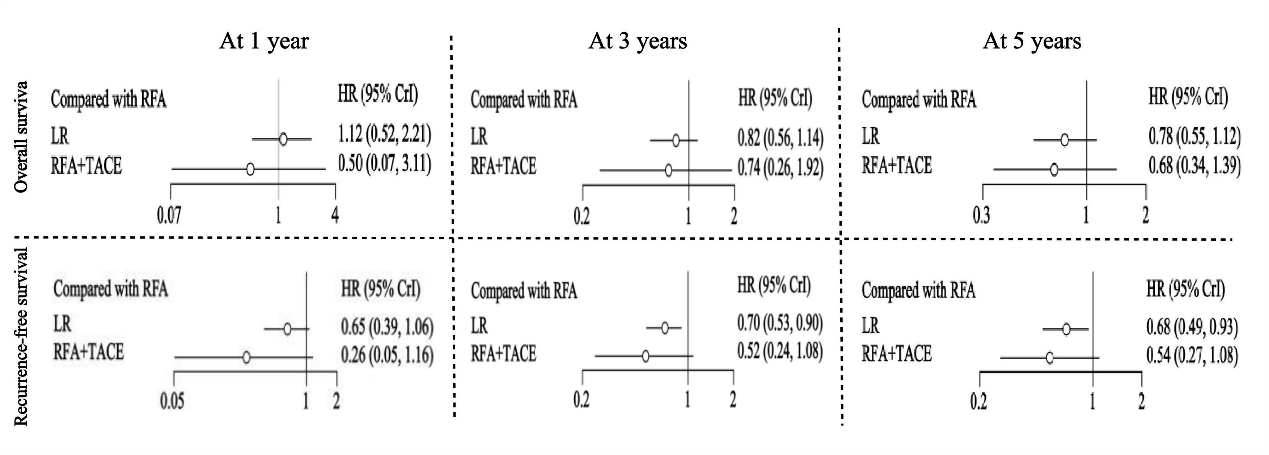


**Supplementary Figure 10.** The inconsistencies of all closed-loop structures are detected by the Node-Splitting method. (Subgroup analysis for the Single tumor studies)


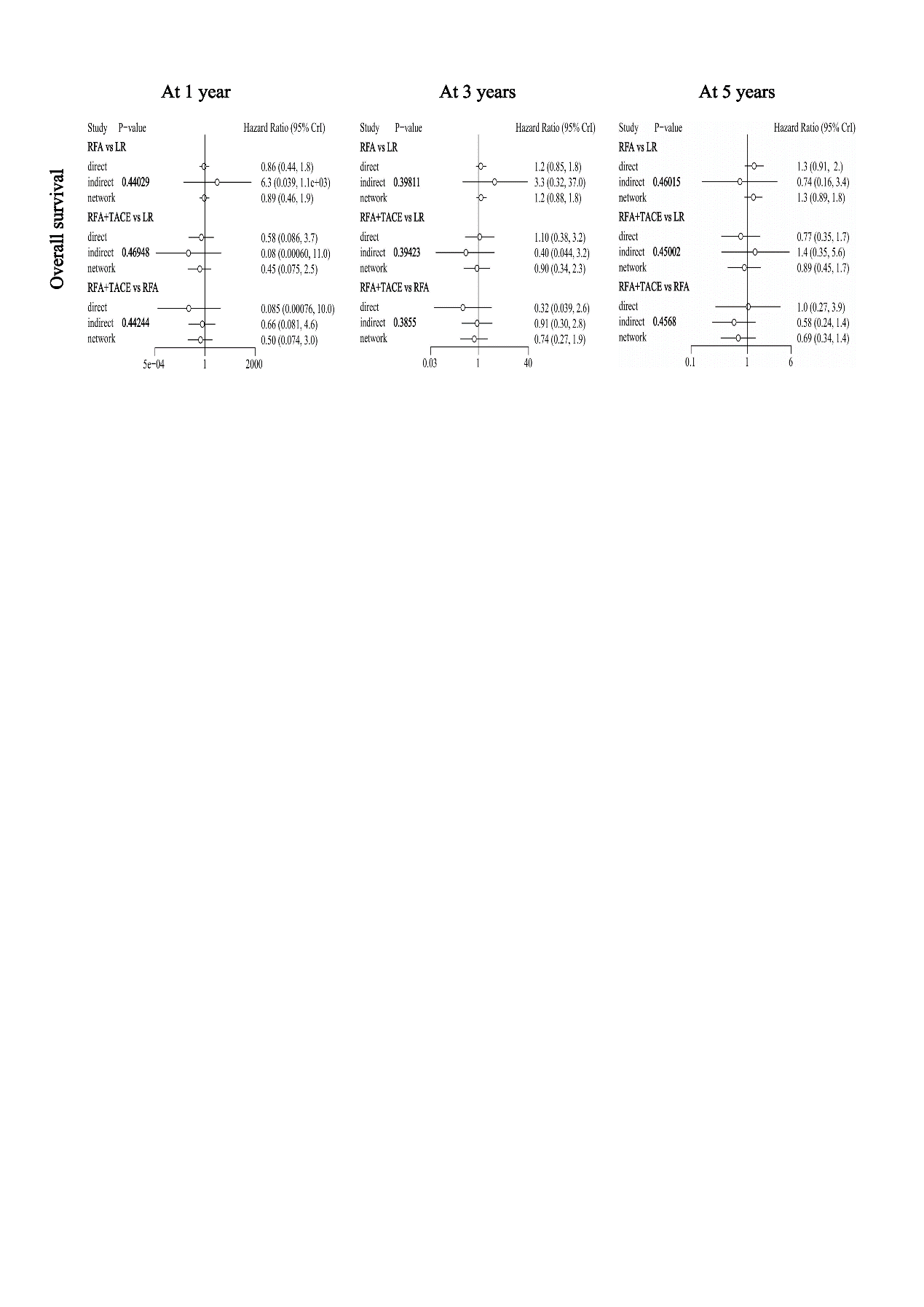


**Supplement Figure 11.** Hazard ratio (for OS and RFS) along with 95% credible interval (CI) for LR and RFA+TACE compared with RFA for the subgroup analysis (Tumor diameter ≤ 3cm).


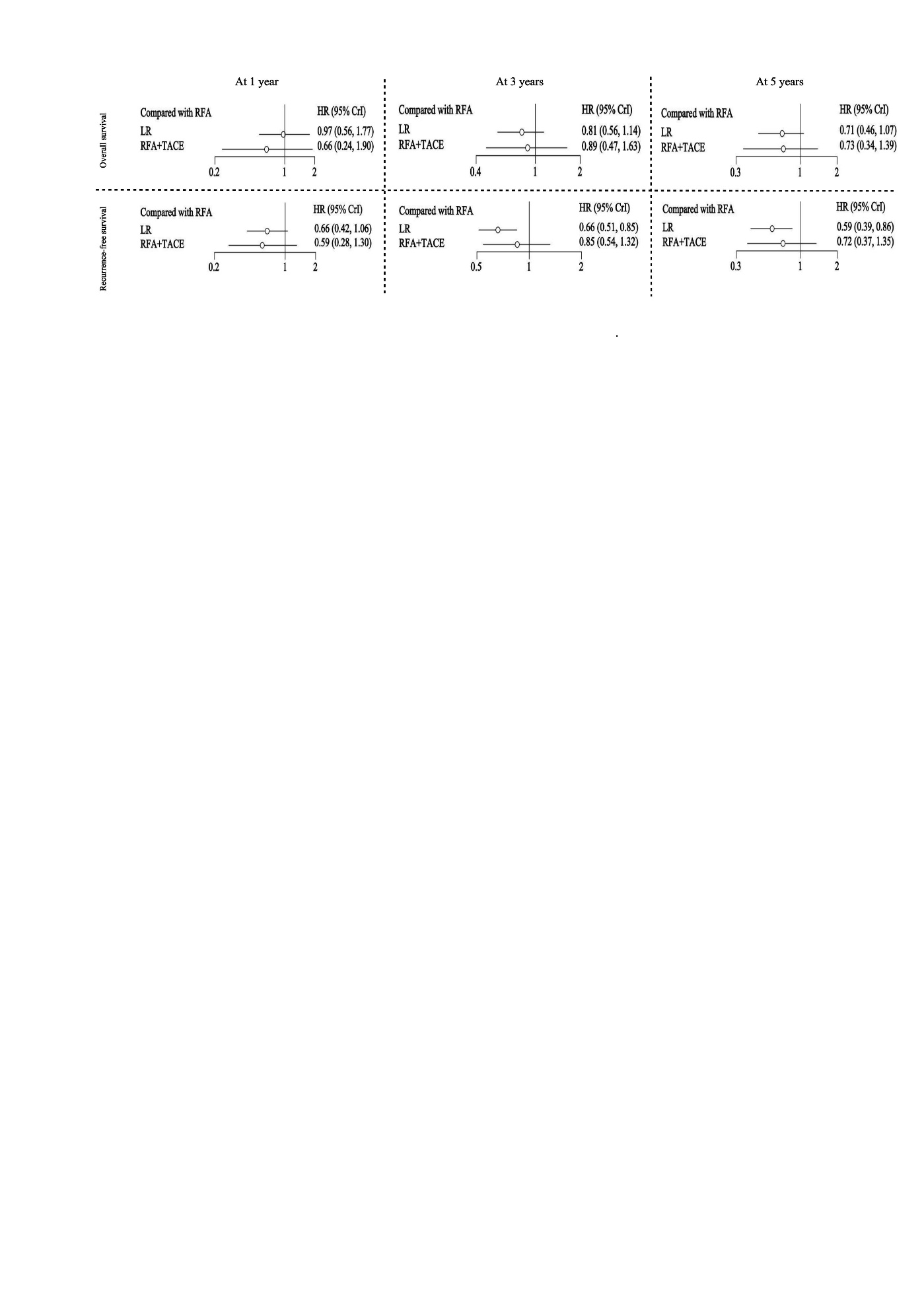


**Supplementary Figure 12.** The inconsistencies of all closed-loop structures are detected by the Node-Splitting method. (Subgroup analysis for the tumor diameter ≤ 3cm)


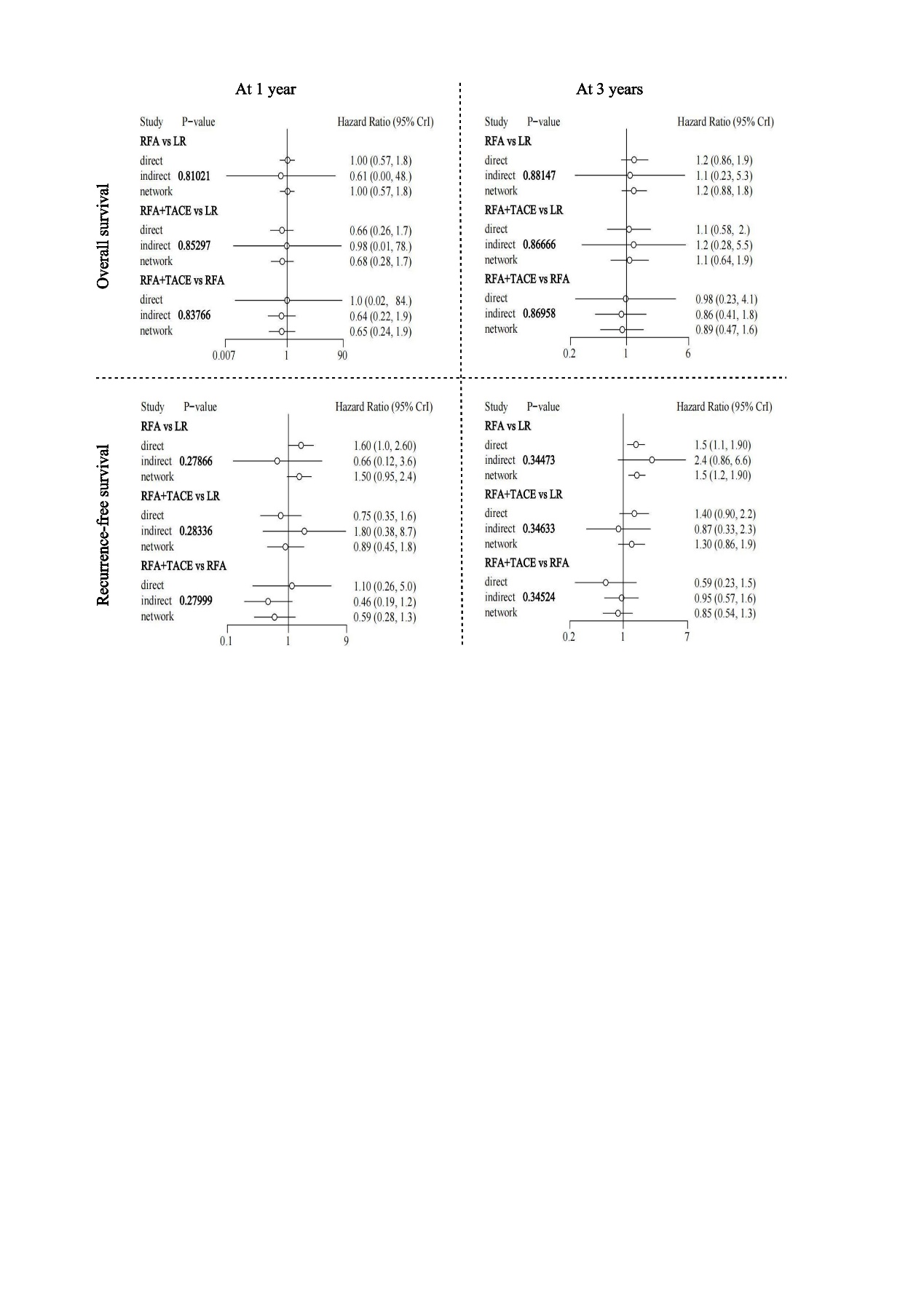

Supplement: Supplementary file 1 [file DataSheet_1.docx]
